# Sterile Immunity to Malaria after DNA Prime/Adenovirus Boost Immunization Is Associated with Effector Memory CD8+T Cells Targeting AMA1 Class I Epitopes

**DOI:** 10.1371/journal.pone.0106241

**Published:** 2014-09-11

**Authors:** Martha Sedegah, Michael R. Hollingdale, Fouzia Farooq, Harini Ganeshan, Maria Belmonte, Yohan Kim, Bjoern Peters, Alessandro Sette, Jun Huang, Shannon McGrath, Esteban Abot, Keith Limbach, Meng Shi, Lorraine Soisson, Carter Diggs, Ilin Chuang, Cindy Tamminga, Judith E. Epstein, Eileen Villasante, Thomas L. Richie

**Affiliations:** 1 US Military Malaria Vaccine Program, Naval Medical Research Center, Silver Spring, Maryland, United States of America; 2 La Jolla Institute for Allergy and Immunology, La Jolla, California, United States of America; 3 Division of Medical, Audio, Visual, Library and Statistical Services, Walter Reed Army Institute of Research, Silver Spring, Maryland, United States of America; 4 USAID, Washington, DC, United States of America; Institut de Recherche pour le Développement, France

## Abstract

**Background:**

Fifteen volunteers were immunized with three doses of plasmid DNA encoding *P. falciparum* circumsporozoite protein (CSP) and apical membrane antigen-1 (AMA1) and boosted with human adenovirus-5 (Ad) expressing the same antigens (DNA/Ad). Four volunteers (27%) demonstrated sterile immunity to controlled human malaria infection and, overall, protection was statistically significantly associated with ELISpot and CD8+ T cell IFN-γ activities to AMA1 but not CSP. DNA priming was required for protection, as 18 additional subjects immunized with Ad alone (AdCA) did not develop sterile protection.

**Methodology/Principal Findings:**

We sought to identify correlates of protection, recognizing that DNA-priming may induce different responses than AdCA alone. Among protected volunteers, two and three had higher ELISpot and CD8+ T cell IFN-γ responses to CSP and AMA1, respectively, than non-protected volunteers. Unexpectedly, non-protected volunteers in the AdCA trial showed ELISpot and CD8+ T cell IFN-γ responses to AMA1 equal to or higher than the protected volunteers. T cell functionality assessed by intracellular cytokine staining for IFN-γ, TNF-α and IL-2 likewise did not distinguish protected from non-protected volunteers across both trials. However, three of the four protected volunteers showed higher effector to central memory CD8+ T cell ratios to AMA1, and one of these to CSP, than non-protected volunteers for both antigens. These responses were focused on discrete regions of CSP and AMA1. Class I epitopes restricted by A*03 or B*58 supertypes within these regions of AMA1 strongly recalled responses in three of four protected volunteers. We hypothesize that vaccine-induced effector memory CD8+ T cells recognizing a single class I epitope can confer sterile immunity to *P. falciparum* in humans.

**Conclusions/Significance:**

We suggest that better understanding of which epitopes within malaria antigens can confer sterile immunity and design of vaccine approaches that elicit responses to these epitopes will increase the potency of next generation gene-based vaccines.

## Introduction

*Plasmodium falciparum* malaria remains a leading cause of morbidity and mortality, especially in children in Africa [1] and developing an effective vaccine is a high priority [2]. CD8+ T lymphocytes are important mediators of protective immunity against the malaria liver stages [3]–[7], killing the intracellular parasites through interferon-gamma (IFN-γ) or release of cytotoxins [8], [9], and thus could provide an effective objective for immunization.

We have pursued a gene-based approach to generate this protective immunity, building on the evidence that heterologous prime-boost immunization induces CD8+ T cells and protection against malaria in mice [10], [11], non-human primates [12] and humans [13]–[20]. Heterologous prime-boost regimens, such as priming with DNA plasmids and boosting with viral vectors, are particularly effective for inducing CD8+ T cells for malaria. We chose the circumsporozoite protein (CSP) and the apical membrane antigen-1 (AMA1) as vaccine antigens for clinical testing, as both are present in sporozoites and liver stages [21], [22] and CSP has induced protective responses against pre-erythrocytic stage malaria in humans [19], [23]. AMA1 is also expressed in blood stages, inducing antibodies associated with protection in malaria-endemic regions [24]. With this approach, we aim to destroy the infection in the liver prior to the release of parasites into the blood, thereby preventing all clinical manifestations of malaria and simultaneously blocking transmission, which requires the development of blood stage infection.

In our first clinical study of a heterologous prime-boost gene-based regimen, four of 15 research volunteers (27%) were fully protected against controlled human malaria infection (CHMI) after receiving three monthly doses of two DNA plasmids encoding CSP and AMA1 (DNA) and a boost four months later with two replication-deficient human adenovirus 5 vectors (Ad) similarly expressing CSP and AMA1 (Ad) (the NMRC-M3V-D/Ad-PfCA Vaccine) [19], [25]. Protection was statistically associated with ELISpot and CD8+ T cell IFN-γ responses to AMA1, but not CSP, providing the first report of a statistically significant association between CD8+ T cell responses and protection in humans [19]. On an individual basis, two of four and three of four protected volunteers had higher activities to CSP and AMA1 respectively, whereas one protected volunteer had low activities to both antigens [19], suggesting that both CSP and AMA1 induced robust responses contributing to protection in some volunteers. In a subsequent trial, designed to test the requirement for DNA priming, a single dose of the Ad vaccine (AdCA), identical to the boost in the DNA/Ad trial, induced strong cellular responses, delayed the onset of parasitemia in one of 18 volunteers, but did not provide sterile immunity in any volunteer [26]. This indicated that DNA priming was essential for protection and implied that the immune correlates identified in the DNA/Ad trial might not hold true for the AdCA trial, since the latter generated robust ELISpot and CD8+ T cell IFN-γ responses that in many volunteers exceeded those induced by the high-responding protected DNA/Ad volunteers [26]. Antibody responses and CD4+ T cell responses in both trials were modest, and showed no association with protection in the DNA/Ad trial [25], [26].

To better understand the immunological responses to DNA/Ad and AdCA, we here compared the cellular responses of the four protected volunteers in the DNA/Ad trial with those of the 11 non-protected volunteers. We then further compared their cellular responses with those of the 18 non-protected volunteers who received the AdCA vaccine without DNA priming to gain insight regarding the mechanisms underlying protection and the beneficial effect of DNA priming. The overarching goal was to identify protective immunological signatures supporting gene-based malaria vaccine design and optimization.

Due to the small number of protected subjects and the limited availability of peripheral blood mononuclear cells (PBMC), we conducted this as an exploratory analysis, aiming to generate hypotheses for future testing. We investigated several parameters reflecting the quality and specificity of T cell activities in the DNA/Ad and AdCA trials. These included: T cell functionality based on production of IFN-γ, IL2 and TNF-α; CD8+ T cell memory differentiation defined by CD45RA and CD27 staining [27]; and antigen-specificity as defined by recognition of discrete regions and epitopes within the two antigens. Our results showed that DNA priming induced T cell activities in three of the four protected volunteers (in two to CSP and three to AMA1) that differed qualitatively from those of non-protected volunteers in the same trial and from all the volunteers in the AdCA trial. We found that, while both DNA/Ad and AdCA regimens induced primarily monofunctional, IFN-γ-secreting CD8+ T cells, the responses in three of the protected volunteers from DNA/Ad trial showed higher effector memory to central memory ratios to AMA1, and one to CSP, whereas volunteers immunized with AdCA generally showed lower effector:central memory ratios even though both effector and central memory responses were robust. In addition, the responses in three of four protected volunteers were narrowly focused on discrete regions of the CSP and AMA1 molecules, whereas AdCA generally induced broader responses targeting multiple regions of each antigen.

These findings led us to hypothesize that, in these limited studies, predominantly monofunctional effector CD8+ T cells that target specific class I-restricted epitopes in vaccine antigens may confer sterile protection to malaria. This appeared to be the case in three of the four protected DNA/Ad volunteers with robust responses to AMA1 and the two of these three who also had robust responses to CSP. One protected volunteer developed low peripheral ELISpot IFN-γ activities to both antigens after Ad boost, although no peripheral CD8+ T cell IFN-γ responses could be identified, suggesting the importance of local (liver) T cells responses in mediating protection [28], [29]. These findings are presented herein and their implications for optimizing vaccine design are discussed.

## Materials and Methods

### Objectives

The objectives of this study were to compare CD8+ T cell activities induced by the DNA/Ad vaccine in protected and non-protected volunteers; to compare CD8+ T cell activities after DNA/Ad immunization with those after AdCA immunization; and to analyze responses to individual CSP and AMA1 peptide pools and individual peptides to determine whether immunized and protected volunteers recognized certain HLA-restricted class 1 epitope(s), using samples collected from the DNA/Ad and AdCA trials. Detailed accounts of each clinical trial have been previously published [19], [26]. The vaccine constructs and CHMI used the 3D7 clone of *P. falciparum*.

### Human ethics statement

The study protocols for these clinical trials were approved by the Institutional Review Boards at the Walter Reed Army Institute of Research (WRAIR) and the National Naval Medical Center (NNMC). The study was conducted at the WRAIR Clinical Trials Center in accordance with: the principles described in the Nuremberg Code and the Belmont Report; all federal regulations regarding the protection of human participants as described in 32 CFR 219 (The Common Rule) and instructions from the Department of Defense, the Department of the Army, the Department of the Navy and the Bureau of Medicine and Surgery of the United States Navy; and the internal policies for human subject protections and the standards for the responsible conduct of research of the US Army Medical Research and Materiel Command (USAMRMC) and the Naval Medical Research Center (NMRC). WRAIR holds a Federal Wide Assurance from the Office of Human Research Protections (OHRP) under the Department of Health and Human Services as does NMRC. NMRC also holds a Department of Defense/Department of the Navy Federal Wide Assurance for human subject protections. All key personnel were certified as having completed mandatory human research ethics education curricula and training under the direction of the WRAIR IRB or the NMRC Office of Research Administration (ORA) and Human Subjects Protections Program (HSPP). All potential study subjects provided written, informed consent before screening and enrollment and had to pass an assessment of understanding.

### Human volunteers

The full details of the clinical findings of these trials, including patient recruitment and flow, safety and tolerability have been previously reported [19], [26]. A total of 33 volunteers were available for this study: 15 subjects were immunized with DNA/Ad and four were fully protected against CHMI [19] and none of the non-protected volunteers showed a significant delay to parasitemia (defined as more than two standard deviations after the geometric mean of the time to parasitemia of control infectivity subjects [30] In a separate trial, 18 subjects were immunized with AdCA, and one showed a significant delay to onset of parasitemia after CHMI but none were fully protected [26].

### Immunological endpoints

DNA/Ad samples were collected pre-DNA immunization, 28 days post the third DNA immunization, 105 days post the third DNA immunization/seven days prior to Ad administration, 22/23 days post Ad administration/five or six days pre-HMI (post-Ad), and four and 12 weeks post CHMI; AdCA samples were collected pre-immunization and 22/23 days post Ad administration/five or six days pre-CHMI;

#### Interferon-gamma Enzyme Linked Immunospot Assays (IFN-γ ELISpot)

T cell responses were measured by IFN-γ ELISpot assay [25] using fresh peripheral blood mononuclear cells (PBMC). Each assay used a bridging volunteer to ensure repeatability between different assays [31]. The full length *P. falciparum* 3D7 CSP sequence was covered by 15 amino acid (aa) peptides overlapping by 11 aa and combined into 9 pools (Cp1-Cp9) each containing three to 12 peptides [25]. Full length AMA1 was covered by 15mers that were combined into 12 pools (Ap1-Ap12) each containing 10–13 peptides [25]. Results, expressed as spot forming cells/million PBMC (sfc/m) are shown as: (1) the magnitude of responses of each volunteer to individual CSP or AMA1 peptide pools, (2) summed responses of each volunteer, or (3) numbers of positive volunteers defined as a volunteer with a positive response to at least one CSP or one AMA1 peptide pool [25], [32]. A positive response to a given CSP or AMA1 peptide pool was defined as positive after showing (1) a statistically significant difference between the number of spot forming cells in triplicate or quadruplicate test wells and triplicate or quadruplicate negative control wells (Student's two tailed *t*-test), plus (2) at least a doubling of spot forming cells in test wells relative to negative control wells, plus (3) a difference of at least ten spots between test and negative control wells. The volunteer was designated as positive when positive against at least one of the pools tested.

#### Flow cytometry with intracellular cytokine staining (ICS)

We have previously reported that four CSP peptide pools (Cp1, Cp2, Cp6 and Cp9) and six AMA1 peptide pools (Ap1, Ap3, Ap4, Ap8, Ap9 and Ap10) were immunodominant using AdCA and AdC-immunized subjects [33]. These selected pools were used in the current study. Frozen PBMC were stimulated by each CSP or AMA1 peptide pools as previously described [25]. Control stimulants were medium alone and the CEF peptide pool (Anaspec, San Jose, CA). Cells were phenotyped as CD4+ and CD8+ T cells and stained for IFN-γ, TNF-α and IL-2. Data for peptide pools were corrected for media response at each time point. A positive response was defined as a frequency of cytokine-stained CD4+ or CD8+ cells exceeding the geometric mean + 3 standard deviations of the medium-stimulated controls, 0.030% [26]. A volunteer was considered positive if activity to one or more peptide pools was at least 0.03%; some volunteers who had summed activities >0.030% were considered negative if activities to individual peptide pools did not reach 0.030%. Samples from each volunteer at each time point were tested in the same assay. Assays of activities of volunteers from the DNA-Ad and AdCA trials were tested separately, but a bridging volunteer was used to ensure repeatability between assays. Activities are shown as (1) each volunteer's responses individual CSP or AMA1 peptide pools, (2) summed responses of all volunteers, or (3) numbers of positive volunteers defined as a volunteer with a positive response to at least one CSP or one AMA1 peptide pool [25], [32]. CD4+ and CD8+ T cell subsets are defined as: IFN-γ monofunctional (IFN-γ^+^IL-2^−^TNF-α^−^), IFN-γ/IL-2 polyfunctional (IFN-γ^+^IL-2^+^TNF-α^−^), IFN-γ/TNF-α polyfunctional (IFN-γ^+^IL-2^−^TNF-α^+^), IFN-γ/IL-2/TNF-α polyfunctional (IFN-γ^+^IL-2^+^TNF-α^+^), IL-2 monofunctional (IFN-γ^−^IL-2^+^TNF-α^−^), IL-2/TNF-α polyfunctional (IFN-γ^−^IL-2^+^TNF-α^+^) or TNF-α monofunctional (IFN-γ^−^IL-2^−^TNF-α^+^).

#### CD8+ T cell memory responses

Frozen PBMC taken at the same time points were stimulated with the immunodominant CSP or AMA1 peptide pools [25] and sorted as CD8+ T cells and phenotyped by CD45RA and CD27 staining as naïve (NV) CD45RA^+^CD27^+^, central memory (CM) CD45RA^−^CD27^+^, effector memory (EM) cells were CD45RA^−^CD27^−^, and terminally differentiated (TD) CD45RA^+^CD27^−^ [34]. Each pool was then stained for IFN-γ, IL-2 or TNF-α and activities and the frequency of NV, TD, CM or EM T cells expressed as per cent of CD8+ T cells. A positive response was defined as exceeding the geometric mean +3 standard deviations of the medium stimulated controls (0.03%). Responses are shown as activities to individual peptide pools, the sum of responses to individual peptide pools, the geometric mean of summed responses, and the number of positive volunteers.

### Statistical analyses

The Mann-Whitney U test was used to compare the summed cellular responses of protected and non-protected volunteers from the two trials. Kruskal-Wallis one-way analysis of variance was used to compare the EM/CM ratios of protected and non-protected volunteers from the two trials. Two-sided tests were used, with p<0.05 considered significant.

## Results

### *Ex vivo* ELISpot, CD8+ T cell, and CD8+ T cell IFN-γ memory activities trend higher in protected than in non-protected volunteers in the DNA/Ad trial

**Figure 1:**
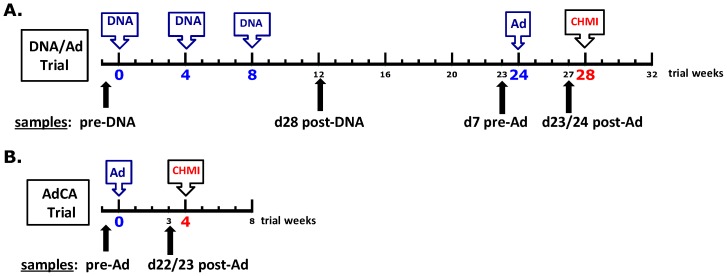
DNA/Ad Trial design. DNA/Ad Trial: Healthy, malaria-naïve adult research subjects received three doses of two DNA plasmids (mixture of 1 mg CSP and 1 mg AMA1, 2 mg total dose, in 2 ml phosphate buffered saline) in two divided doses of 1 ml into each deltoid muscle by needleless jet injection, followed by one dose of two adenovectors (mixture of 1×10^10^ particle units CSP and 1×10^10^ particle units of AMA1, 2×10^10^ particle units total dose, in 1 ml final formulation buffer) into one deltoid muscle by needle and syringe. Both Ad5 seropositive and seronegative study subjects were enrolled. AdCA Trial: Malaria-naïve adult research subjects received one dose of two adenovectors (formulated identically as in DNA/Ad) into one deltoid muscle by needle and syringe. Only Ad5 seronegative study subjects (neutralizing antibody titer <500, NVITAL assay) were enrolled. CHMI  =  controlled human malaria infection, administered via the bites of five laboratory-reared *P. falciparum*-infected *Anopheles stephensi* mosquitoes.

We first compared T cell activities of protected and non-protected volunteers in the DNA/Ad trial measured by IFN-γ ELISpot assay and flow cytometry/intracellular cytokine staining (ICS), considering both the magnitude of the responses and whether or not responses were positive by pre-set criteria (see Methods). The trial design and sampling time points for DNA/Ad, as well as the AdCA trial, are provided in Figure 1.

### Ex vivo ELISpot IFN-γ activities

We previously reported the geometric mean of CSP- and AMA1-specific ELISpot IFN-γ activities of all 15 (protected and non-protected) volunteers, based on the summed responses of each volunteer following stimulation of peripheral blood mononuclear cells (PBMCs) with nine CSP or 12 AMA peptide pools spanning the full length of each antigen [19]. Here, we show activities to individual peptide pools, and the geometric mean and range of the 11 non-protected volunteers and the individual activities of each protected volunteer (v6, v10, v11, v18).

#### CSP

**Figure 2:**
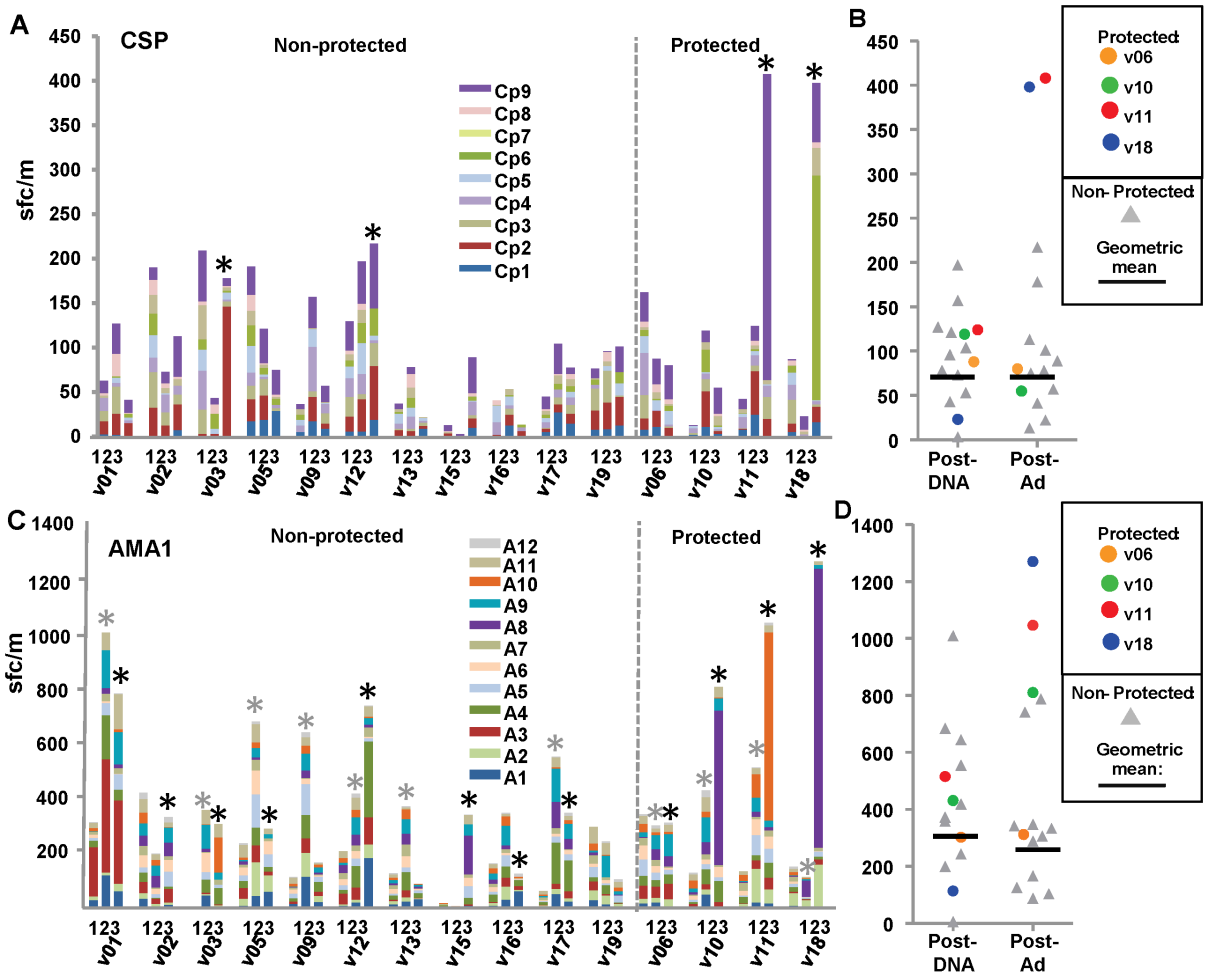
DNA/Ad ELISpot IFN-γ activities to CSP and AMA1 peptide pools. Panels A and C: ELISpot activities (sfc/m) of each volunteer to CSP or AMA1 peptide pools are shown as color coded stacked bars at pre-immunization (1), 28 days after DNA immunization (2) and 22/23 days after the Ad boost (3). *Positive activities. Panels B and D: ELISpot activities were summed and protected subjects shown as color-coded dots. Horizontal bars represent geometric mean activities of non-protected volunteers. The geometric means of summed activities of non-protected volunteers and the activities of each protected volunteer are shown in Table 1. Panel A: CSP: All volunteers were negative after DNA immunization, but four volunteers were positive after Ad boost including protected v11 (408 sfc/m) and v18 (398 sfc/m) that were above non-protected volunteers, whereas protected v06 and v10 were negative. Most activity of v11 and v18 was directed to single CSP peptide pools. Panel B: CSP: Geometric means of non-protected volunteers remained similar after DNA immunization and Ad boost when v11 and v18 were higher than all other volunteers. Panel C: AMA1: 11 volunteers were positive after DNA immunization (*gray), and 11 were positive after Ad boost (*black), including protected v10 (810 sfc/m), v11 (1046 sfc/m) and v18 (1270 sfc/m) that were above non-protected volunteers, whereas protected v06 was within the range of non-protected volunteers. Most activity of v10, v11 and v18 was directed to single AMA1 peptide pools. Panel D: AMA1: Geometric means of non-protected volunteers were higher Ad boost when v10, v11 and v18 higher than all volunteers.

After DNA immunization, no volunteers were positive (Figure 2, Panel A) and the geometric mean of summed ELISpot responses in non-protected volunteers was 68 sfc/m (range 3–197 sfc/m) (Figure 2, Panel B). Four months later, after the Ad boost, 4–5 days before CHMI, two non-protected and two protected volunteers (v11 and v18) were positive (Figure 2, Panel A), and the geometric mean remained low in non-protected volunteers (68 sfc/m, range 13–217 sfc/m) (Figure 2, Panel B, Table 1). Although ELISpot responses to CSP were not associated with protection as assessed in our prior study [19]), activities of v11 and v18 were higher than all non-protected volunteers. Protected v11 and v18 predominantly recognized single CSP peptide pools (Cp9 and Cp6, Figure 2, Panel A).

**Table 1:** Cell mediated activities in the DNA/Ad and AdCA trials at post-Ad time point (4–5 days before challenge).

	ELISpot sfc/m		ICS/Flow cytometry					
			CD8+ %		CD8+ CM %		CD8+ EM %	
	CSP	AMA1	CSP	AMA1	CSP	AMA1	CSP	AMA1
**A. DNA/Ad**								
**Protected volunteers**								
v06	80	**312**	<0.03	0.08	<0.03	0.04	<0.03	<0.03
v10	55	**810**	<0.03	0.22	<0.03	0.09	<0.03	**0.07**
v11	**408**	**1046**	**0.21**	**0.98**	**0.08**	**0.21**	**0.05**	**0.46**
v18	**398**	**1270**	**0.10**	**0.52**	**0.06**	**0.17**	<0.03	**0.27**
No. positive^1^/total number	2/4	4/4	2/4	3/4	2/4	3/4	1/4	3/4
**Non-protected volunteers**								
Geometric mean	68	262	0.04	0.11	<0.03	0.05	<0.03	<0.03
Range	13–217	88–787	<0.03–0.29^3^	0.05–0.19	<0.03–0.13^3^	<0.03–0.16	<0.03–0.03	<0.03–0.07
No. positive^1^/total number	2/11	8/11	2/11	8/11	2/11	5/11	0/11	0/11
No. with higher activity than highest protected^2^	0/11	0/11	1^3^/11	0/11	1^3^/11	0/11	0/11	0/11
**B. AdCA Non-protected**								
Geometric mean	273	1303	0.12	0.49	0.05	0.31	<0.03	0.08
Range	38–2550	435–4594	0.05–0.57	0.13–2.44	0–0.38	0.08–1.54	<0.03–0.09	<0.03–0.33
No. positive^1^/total number	14/18	18/18	13/16	16/16	8/16	15/16	3/16	10/16
No. with higher activity than highest protected^2^	5/18	11/18	5/16	2/16	5/16	11/16	3/16	0/16^4^
**C. Comparisons of geometric mean activities of non-protected volunteers**								
DNA/Ad Non-protected: ADCA Non-protected p value^5^	0.0018	<0.0001	0.0069	0.0002	0.0025	0.0001	0.0500	0.0014

1 A volunteer was considered positive for an assay if activity to one or more CSP or AMA1 peptide pools was positive (see Methods). For individual protected volunteers, positive activities are in bold.

2 The number of volunteers with values higher than the highest value among the four protected DNA/Ad volunteers.

3 Highest values are for v15.

4 Two protected volunteers (v10 and v18) were lower than the highest AdCA activity.

5 p value: non-protected volunteers were included in the comparison. Statistical comparisons are included as an exploratory analysis, since the different trials were not designed or powered for a formal statistical comparison. No.  =  Number.

#### AMA1

After DNA immunization, seven non-protected and all four protected volunteers were positive (Figure 2, Panel C), and the geometric mean of summed ELISpot responses in non-protected volunteers was 295 sfc/m (range 6–1009 sfc/m) (Figure 2, Panel D). Four months later, after the Ad boost, eight non-protected and all four protected volunteers remained positive (Figure 2, Panel C), and the geometric mean of non-protected volunteers remained similar (262 sfc/m, range 88–787 sfc/m) (Figure 2, Panel D, Table 1). ELISpot responses to AMA1 were associated with protection as assessed in our prior study [19], and three protected volunteers (v10, v11, v18) showed higher ELISpot activities than all non-protected volunteers. Protected v10, v11 and v18 predominantly recognized single AMA1 peptide pools (v10 and v18, Ap8; v11, Ap10) (Figure 2, Panel C). In summary, high IFN-γ ELISpot responses in two of four protected volunteers to CSP and three of four protected volunteers to AMA1, predominantly to single peptide pools, may have contributed to protection in the DNA/Ad trial.

### Flow cytometry CD8+ T cell IFN-γ activities

We previously reported the geometric mean of CD8+ T cell IFN-γ activities of all 15 volunteers following stimulation with a single CSP or AMA1 megapool that contained all 65 CSP or all 153 AMA1 15mer peptides [19]. Here, we report the summed CD8+ T cell IFN-γ activities to individual peptide pools, but were able to test only four of nine CSP and six of 12 AMA1 pools used in the ELISpot IFN-γ assays, due to limited PBMC availability. These 10 pools were selected because of immunodominance in prior studies, where the majority of IFN-γ responses were recalled by these pools [25], [32], [33]. However, summed activities using individual peptide pools were greater than those used the single megapool, and this may reflect decreased competition for MHC-binding sites, or differences in concentration of individual 15mer peptides within these pools. We compared the geometric mean and range of the 11 non-protected volunteers with the activities of each protected volunteer (v6, v10, v11, v18).

#### CSP

**Figure 3:**
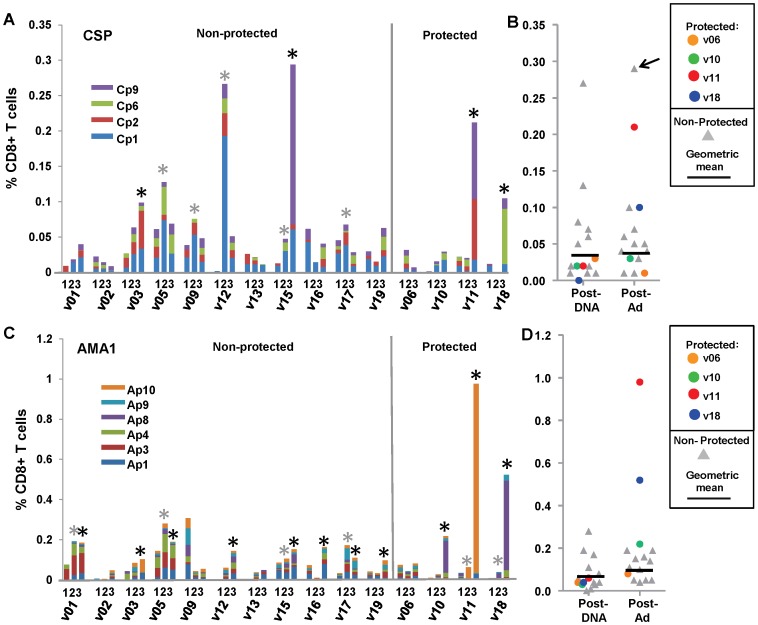
DNA/Ad CD8+ T cell IFN-γ activities to CSP and AMA1 peptide pools. Panels A and C: CD8+ T cell IFN-γ activities of each volunteer to CSP or AMA1 peptide pools are shown as color coded stacked bars at pre-immunization (1), 28 days after DNA immunization (2) and 22/23 days after the Ad boost (3). *Positive activities (gray: post-DNA: black: post-Ad). Panels B and D: CD8+ T cell IFN-γ activities were summed and are shown as color-coded dots. Horizontal bars represent geometric mean activities of non-protected volunteers. The geometric means of summed activities of non-protected volunteers and the activities of each protected volunteer are shown in Table 1. Panel A: CSP: Five non-protected volunteers were positive after DNA immunization, but four volunteers were positive after Ad boost including protected v11 (0.21%) and v18 (0.10%) that were above non-protected volunteers, except v15 that had highest activity (0.29%), whereas protected v06 and v10 were negative. Most activity of v11, v18 and v15 was directed to single CSP peptide pools. Panel B: CSP: Geometric means of non-protected volunteers remained similar after DNA immunization and Ad boost when v11 and v18 were higher than all other volunteers, except v15 (arrow). Panel C: AMA1: Four non-protected volunteers were positive after DNA immunization, and 11 were positive after Ad boost, including protected v10 (0.22%), v11 (0.98%) and v18 (0.52%) that were above non-protected volunteers, whereas protected v06 negative. Most activity of v10, v11 and v18 was directed to single AMA1 peptide pools. Panel D: AMA1: Geometric means of non-protected volunteers rose after Ad boost compared to after DNA immunization, and v10, v11 and v18 higher than all volunteers.

After DNA immunization, the geometric mean of summed CD8+ T cell activities in non-protected volunteers was low (0.04%, range <0.03–0.27%) (Figure 3, Panel C), five volunteers were positive, while no protected volunteers were positive. After the Ad boost, only two non-protected volunteers were positive (v03 and v15) resulting in a geometric mean of 0.04% (range <0.03–0.29%) (Table 1). Activities of two protected volunteers pre-challenge became positive (v11, v18) and were higher than all non-protected volunteers (except non-protected v15, who had the highest activities post Ad). Volunteer v11 predominantly recognized the single peptide pool Cp9, and v18 predominantly recognized a single peptide pool, Cp6 (Figure 3, Panel A and B).

#### AMA1

After DNA immunization, the geometric mean of summed CD8+ T cell activities in non-protected volunteers was low (0.05%, range <0.03–0.28%) (Figure 3, Panel D), and four non-protected volunteers were positive. After the Ad boost, eight non-protected and three protected volunteers were positive (Figure 3, Panel C), and the geometric mean of non-protected volunteers increased (0.11%, range 0.05–0.19%) (Table 1). Activities of three protected volunteers (v10, v11, v18) were higher than those of all non-protected volunteers. These three protected volunteers predominantly recognized single peptide pools (v10 and v18: Ap8; v11: Ap10) (Figure 3, Panel C and D). The IFN-γ producing-CD8+ T cell responses to CSP and AMA1 thus mirrored the IFN-γ ELISpot results, and suggest that CD8+ T cell responses are an important component of the cell populations assessed by the ELISpot assay [25].

### Flow cytometry CD8+ T cell IFN-γ memory activities

**Figure 4:**
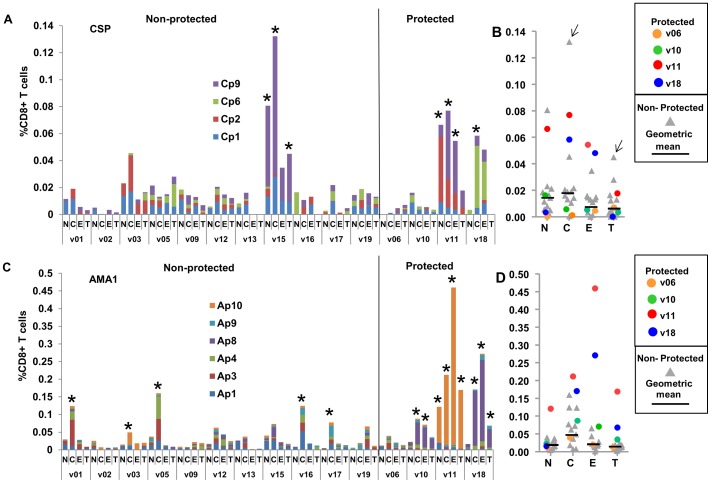
DNA/Ad CD8+ T cell memory IFN-γ activities to CSP and AMA1 peptide pools. Panels A and C: CD8+ T cell IFN-γ activities of each volunteer to CSP or AMA1 peptide pools 22/23 days after the Ad boost are shown as color coded stacked bars, differentiating memory naïve (N), central (C), effector (E) and terminally differentiated (T) cells (per cent of CD8+ T cells) to CSP and AMA1 peptides as color-coded bars. Non-protected and protected volunteers are grouped. *Positive activities (defined in Methods). Panels B and D: CD8+ T cell IFN-γ memory activities were summed and are shown as color-coded dots. Horizontal bars represent geometric mean activities of non-protected volunteers. The geometric means of summed activities of non-protected volunteers and the activities of each protected volunteer are shown in Table 1. Panel A: CSP: among non-protected volunteers, only one (v15) had positive CM and TD activities and none had positive EM activities. One protected volunteer (v11) had positive CM, EM and TD activities whereas protected v18 had only positive CM activity. Positive CM, EM and TD activities were predominantly directed to single CSP peptide pools. Panel B: the geometric mean of CM and EM activities of non-protected volunteers were lower than protected v11 and v18, except CM activity of non-protected v15 (arrow). Panel C: AMA1: five non-protected volunteers only had positive CM activities, whereas protected v10 had positive CM and EM, and v11 and v18 had positive CM, EM and TD activities that were mostly directed to single AMA1 peptide pools. Panel D: AMA1: the geometric mean of CM activities of non-protected volunteers was similar to v10 but lower than v11 and v18; however, protected v10, v11 and v18 were above the geometric means of EM and TD activities of non-protected volunteers.

We next examined the activities of naïve (NV), central memory (CM), effector memory (EM) and terminally differentiated (TD) CD8+ T cells after the Ad boost, following stimulation with the selected peptide pools and summing responses. Again we compared the individual activities of the four protected volunteers to the geometric mean and range of the 11 non-protected volunteers (Figure 4).

#### CSP

After the Ad boost, among non-protected volunteers, only v15 had positive NV, CM and TD activities and none including v15 developed positive EM activity (Figure 4, Panel A). Geometric mean NV, CM, EM and TD activities were all <0.03% (Figure 4, Panel B) and EM activities are shown in Table 1. Among protected volunteers, only v11 developed positive CM, EM and TD activities and v18 developed only positive CM activity (Figure 4, Panels A and B). Volunteer v11 EM activity predominantly recognized Cp9.

#### AMA1

After the Ad boost, five non-protected volunteers had positive CM activities, but none had positive EM or TD activities (Figure 4, Panel C). Three protected volunteers, v10, v11 and v18, also developed positive CM activities, as well as EM and TD activities that were absent in all non-protected volunteers (Figure 4, Panels C and D). Three of the four protected volunteers developed CM, EM and TD activities that predominantly recognized Ap8 (v10 and v18) and Ap10 (v11). The fourth protected volunteer (v06) did not have positive CM, EM or TD activities against CSP nor AMA1.

### *Ex vivo* ELISpot, CD8+ T cell, and CD8+ T cell memory IFN-γ activities after AdCA equal or exceed those after DNA/Ad

Since DNA priming is known to affect subsequent responses to an Ad boost [35], we examined T cell activities after immunization with the AdCA vaccine, in the absence of DNA priming, to see if the same protection-associated immune profiles identified in the DNA/Ad trial (high IFN-γ ELISpot, CD8+ T cell IFN-γ, and CD8+ T cell IFN-γ EM activities) were also induced in the AdCA trial. Because anti-adenovirus-5 neutralizing antibodies (NAb) may affect immune responses to Ad5-vectored vaccines [36], we were concerned that differing enrollment criteria might affect the validity of this comparison, since research subjects with pre-existing NAb were enrolled into the DNA/Ad but not the AdCA trial. However, NAb had no significant effect on any T cell activities reported here after DNA/Ad immunization (p = >0.05) supporting the validity of comparison of T cell responses from both trials in this report. We compared the *ex vivo* ELISpot, CD8+ T cell, and CD8+ T cell CM and EM IFN-γ activities after DNA/Ad with those after AdCA. Table 1 summarizes results for the four protected volunteers from DNA/Ad, the non-protected volunteers from DNA/Ad, and the non-protected volunteers from AdCA, including how many met positivity criteria for each assay, and geometric means and ranges for each group. More detailed cellular response data from the AdCA clinical trial are presented in Figures S1, S2, and S3 (ELISpot, CD8+ T cell, and CD8+ T cell memory IFN-γ activities).

Although higher activities occurred in protected compared with non-protected volunteers in DNA/Ad, unexpectedly the activities of the non-protected volunteers from AdCA were similar to or higher than those of the protected volunteers in the DNA/Ad trial, including ELISpot IFN-γ (Figure S1), CD8+ T cell (Figure S2) and CD8+ T cell EM IFN-γ (Figure S3) responses to CSP and AMA1 (Table 1). For example, 11/18 non-protected volunteers from AdCA had higher IFN-γ ELISpot responses than any of the four protected volunteers from DNA/Ad, 2/16 had higher IFN-γ CD8+ T cell responses, and for CD8+ T cell EM responses, while none were higher than the highest responses in the protected volunteers in DNA/Ad, the overall ranges of activities and percent positive were similar. In the case of CD8+ T cell CM IFN-γ activities, 5/16 and 11/16 AdCA volunteers to CSP and AMA1 respectively were higher than those of the four protected volunteers in DNA/Ad (Table 1). Also strikingly, activities in the non-protected AdCA volunteers were generally much more robust than those of the non-protected volunteers from DNA/Ad, and this difference was statistically significant for all measures (Table 1). We conclude that, while ELISpot, CD8+ T cell and CD8+ T cell EM IFN-γ activities were higher in protected than non-protected volunteers in the DNA/Ad trial, many non-protected volunteers in AdCA demonstrated activities that were as high as or higher than those of the protected volunteers in DNA/Ad. In contrast to the protected volunteers in the DNA/Ad trial, activities in the AdCA trial were predominantly directed to multiple antigen peptide pools. We suggest that DNA-priming may have dampened the Ad-induced activities against specific regions of the two vaccine antigens in the non-protected volunteers in the DNA/Ad trial when compared to those in the AdCA trial. If this is the case, ELISpot, CD8+ T cell and CD8+ T cell EM IFN-γ responses against CSP and AMA1 might not differentiate between protected and non-protected volunteers when the two trials were considered together. This consideration formed the rationale for a more detailed investigation of (1) the quality (as characterized by cytokine functionality) of the T cell responses and (2) identification of their epitope targets in the DNA/Ad and AdCA trials.

### Quality of CD8+ T cell activities induced by both DNA/Ad and AdCA immunization are predominantly IFN-γ monofunctional with lower polyfunctional and TNF-α and IL-2 monofunctional activities

**Figure 5:**
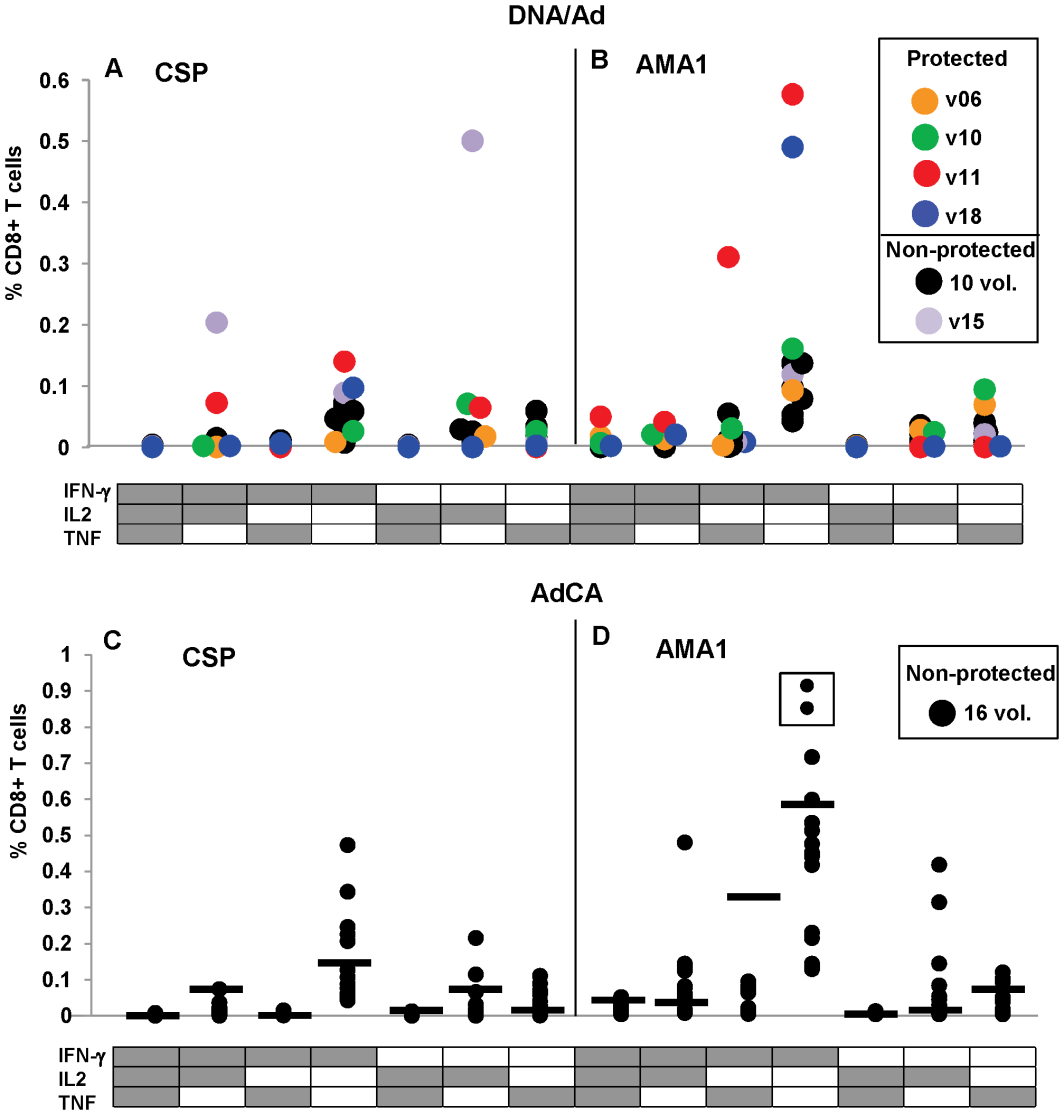
DNA/Ad and AdCA CD8+ T cell antigen-specific activities are predominantly monofunctional with lower polyfunctional responses. Monofunctional and polyfunctional CD8+ T cell activities to CSP and AMA1 after DNA/Ad (Panels A and B) and AdCA (Panels C and D) immunization are shown as color-coded filled circles that represent the percent of CD8+ T cells containing cytokine(s). Black horizontal bar denotes highest activities in DNA/Ad compared with AdCA activities. Activities to individual CSP and AMA1 peptide pools are shown in Figures S1 and S2. Panel A: DNA/Ad CSP: IFN-γ monofunctional activities of protected v11 and v18 were higher (v11, 0.14%; v18, 0.1%) than the two positive non-protected volunteers (v03, 0.07%; v15, 0.09%). Non-protected v15 developed the highest IFN-γ/IL2 polyfunctional (0.20%) and IL2 monofunctional (0.5%) activities. Protected v11 developed positive IFN-γ/IL2 polyfunctional (0.07%) and IL2 monofunctional (0.06%) activities. Panel B: DNA/Ad AMA1: IFN-γ monofunctional activities of protected v10, v11 and v18 (v10, 0.16%; v11, 0.58%, v18, 0.49%) were higher than the eight non-protected volunteers (range 0.05–0.14%). Non-protected v17 and protected v10 also developed lower TNF-α monofunctional (0.07%, 0.09%) activity; v11 also developed IFN-γ/TNF-α polyfunctional (0.31%) activity and lower IFN-γ/IL2 polyfunctional (0.04%), and IFN-γ/IL2/TNF-α polyfunctional (0.05%) activities. Panel C: AdCA CSP: 11 volunteers developed IFN-γ monofunctional activities (0.05%–0.48%), of whom one volunteer developed IFN-γ/IL2 polyfunctional, three developed IL2 monofunctional and two additional volunteers also developed IL2 monofunctional activities. Four volunteers had higher IFN-γ and two volunteers had higher IL2 monofunctional activities than protected volunteers in DNA/Ad. Panel D: AdCA AMA1: all 16 volunteers developed positive IFN-γ monofunctional – boxed volunteers are above scale (1.60% and 1.79% respectively), of whom nine developed lower IFN-γ/IL2 polyfunctional or IFN-γ/TNF-α polyfunctional activities; three volunteers developed IL2 monofunctional and TNF-α monofunctional activities. Four volunteers had higher IFN-γ monofunctional and eight volunteers had higher IFN-γ/IL2 polyfunctional activities than protected volunteers in the DNA/Ad trial.

We first examined the quality of CD8+ T cell activities defined by the presence of IFN-γ, IL-2 and TNF-α using ICS. Polyfunctional CD8+ T cells have been identified as a correlate of protection induced in mice by an adenovirus/modified vaccinia virus Ankara(MVA) malaria vaccine [37], but it is unclear if these findings apply to human CD8+ T cell responses against prime-boost malaria vaccines. Figure 5 shows the summed mono- and polyfunctional CD8+ T cell responses (defined by IFN-γ, IL-2 and TNF-α) for each trial.

### CD8+ T cell IFN-γ activities

#### CSP

In the DNA/Ad trial, after the Ad boost, (Figure 5, panel A), the highest summed activities against CSP of two of the four protected volunteers, v11 and v18, were IFN-γ monofunctional CD8+ T cells; v11 also developed IFN-γ/IL-2 polyfunctional and IL-2 monofunctional activities. Non-protected v03, and v15 (who had overall high summed CD8+ T cell activity predominantly to a single pool, Cp9) developed CD8+ T cell monofunctional IFN-γ activities that were slightly lower than v18; v15 also developed higher IL-2 monofunctional and IFN-γ/IL-2 polyfunctional activity than v11. In the AdCA trial (Figure 5, panel C), 11/16 volunteers developed predominantly IFN-γ monofunctional activities as did protected v11 and v18, and five of these showed activities that were higher than either protected volunteer. One of these also developed similar IFN-γ/IL-2 activity; two of these also developed higher IL-2 and concurrently developed TNF-α monofunctional activity; and two volunteers only developed positive TNF-α activity.

#### AMA1

In the DNA/Ad trial, after the Ad boost, (Figure 5, panel B), the highest summed activities for three of the four protected volunteers, v10, v11 and v18, were also IFN-γ monofunctional CD8+ T cells. In addition, v10 developed lower levels of TNF-α monofunctional activity, and v11 developed IFN-γ/TNF-α polyfunctional activity as well as lower levels of IFN-γ/IL-2/TNF-α and IFN-γ/IL-2 polyfunctional activities. Seven/11 non-protected volunteers only developed lower IFN-γ monofunctional activities, except one who also developed lower TNF-α monofunctional activity. In the AdCA trial (Figure 5, panel D), 16/16 volunteers developed IFN-γ monofunctional activity and 12/16 had higher activities than those of the lowest positive protected volunteer (v10), seven/16 were higher than protected v18, and four/16 were higher than v11. Nine/16 volunteers developed lower levels of IFN-γ/IL-2 polyfunctional, five/16 developed IFN-γ/TNF polyfunctional, and five/16 developed TNF-α monofunctional activities.

In summary, the most frequent recall summed responses in protected v10, v11 and v18 were from IFN-γ monofunctional CD8+ T cells, especially to AMA1, and these were always more robust than responses in non-protected volunteers from the DNA/Ad trial. Polyfunctional responses were less prominent in both protected and non-protected volunteers (excepting v15's response to CSP). In contrast to non-protected volunteers from DNA/Ad, non-protected volunteers from AdCA showed IFN-γ monofunctional activities that were equal to or higher than those recorded for protected v10, v11 and v18 in the DNA/Ad trial. Therefore neither the frequency of CD8+ T cell IFN-γ monofunctional responses nor monofunctional or polyfunctional activities to other cytokines appeared to correlate with protection across both trials. Interestingly, one protected volunteer (v11) developed IFN-γ/TNF-α polyfunctional activity to AMA1 that was not induced by AdCA immunization, while non-protective AdCA immunization induced IL-2 monofunctional and IFN-γ/IL-2 polyfunctional activities to AMA1 in a few volunteers that were not induced by DNA/Ad.

We also examined the CD8+ T cell CM, EM and TD memory subsets for IFN-γ, IL-2 and TNF-α (Figures S4 and S5 for CSP and AMA1, respectively). Positive TD responses were too infrequent to draw conclusions, but CM and EM functionality by IFN-γ, IL-2 and TNF-α activities reflected the general findings described above: the IFN-γ monofunctional phenotype predominated, but there were also some polyfunctional responses, especially for the CM subpopulation, and there was no evident correlate of protection across both trials. After AdCA immunization, CM, EM and TD activities were almost entirely IFN-γ monofunctional, with low IL-2 and TNF-α, characteristic of T cell exhaustion [27], [38].

### Some protected volunteers show higher EM/CM ratios compared with non-protected volunteers from DNA/Ad or AdCA clinical trials

**Figure 6:**
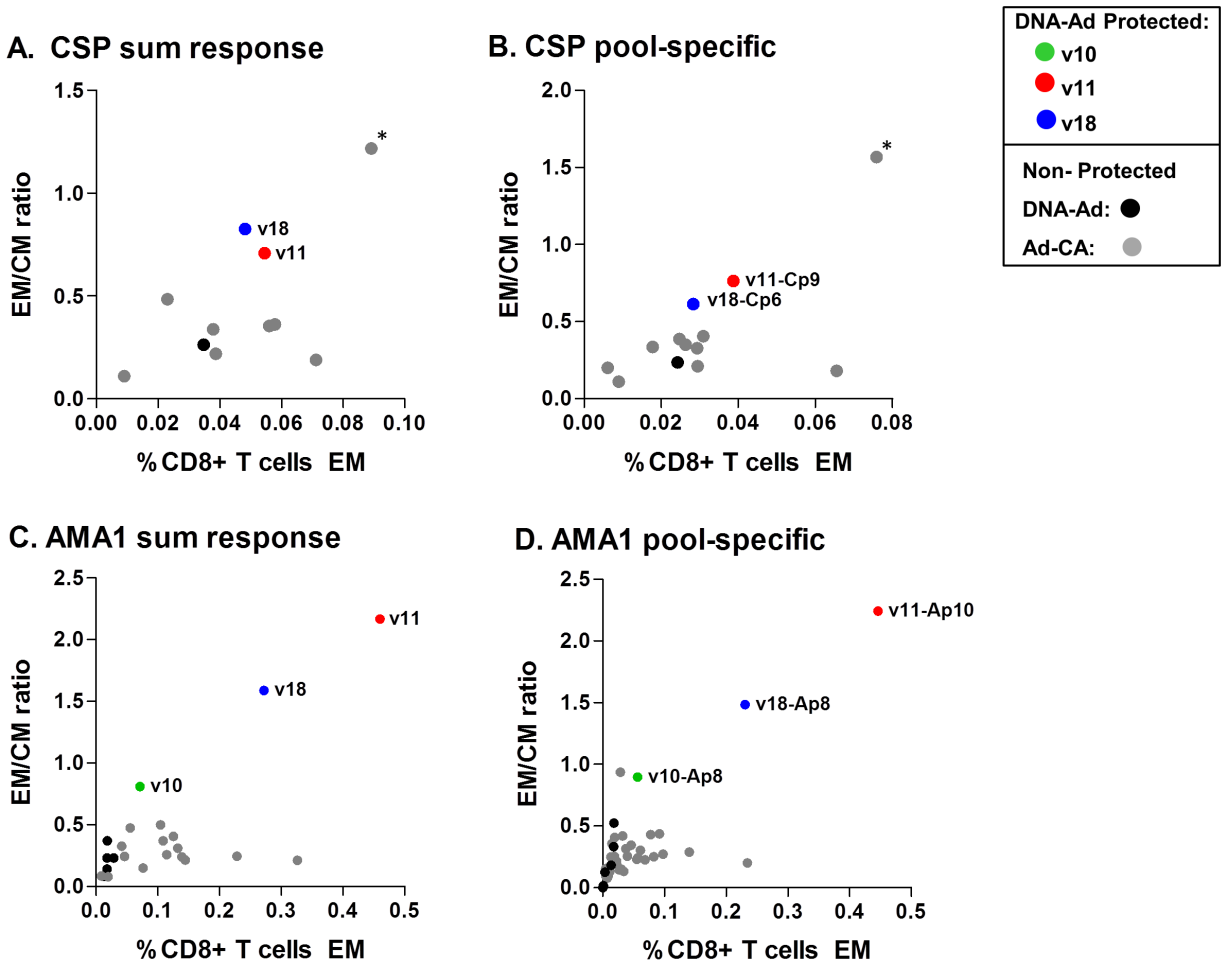
Ratios of effector to central memory in DNA/Ad and AdCA trials for CSP and AMA1 (based on summed and pool-specific responses). All volunteers who had positive summed and pool-specific CD8+ IFN-γ memory central (CM) and effector (EM) activities after Ad boost for CSP or AMA1 were plotted as the ratio of EM:CM vs. EM activity. In addition, some volunteers who had positive CM but negative EM activities were included. Volunteers with negative CM and EM activities were not included. Panel A. CSP summed response: protected v11 and v18 had a higher EM:CM ratio than all other volunteers in the DNA/Ad and AdCA trials, except v135 in the AdCA trial (*). EM activity of v18 did not meet the positivity definition. Panel B. CSP pool-specific response: protected v11 and v18 had a higher EM:CM ratios against Cp9 (v11) and Cp6 (v18) respectively than all other volunteers with positive EM and/or CM activities against any individual pools in the DNA/Ad and AdCA trials, except v135 in the AdCA trial. EM activity of v18 did not meet the positivity definition. Panel C. AMA1 summed response: protected v10, v11, and v18 had a higher EM:CM ratio than all other volunteers with positive EM and/or CM activities in the DNA/Ad and AdCA trials. Panel D. AMA1 pool specific response: protected v10, v11 and v18 had a higher EM:CM ratios against Ap8 (v10 and v18) and Ap10 (v11) compared to all other volunteers in the DNA/Ad and AdCA trials.

We observed that the ratio of EM:CM activities (either summed activities, or activities to the immunodominant pool) appeared to differentiate some protected from non-protected volunteers in the DNA/Ad and AdCA trials for both CSP and AMA1 (Figure 6). We included all volunteers with positive CM and EM activities as well as volunteers who had positive EM or CM activities. Protected v11 (Ap10), v10 and v18 (Ap8) had higher EM:CM ratios to AMA1 than non-protected volunteers across the two trials; similarly, protected v11 (Cp9) had higher EM:CM ratios to CSP than non-protected volunteers, except non-protected v135 (Cp9). We hypothesize that protection requires both a threshold of EM responses, and also a high EM:CM ratio, suggesting that other characteristics of the EM cells were not measured.

In summary, based on positive summed responses against each of the two antigens, the functionality of the CD8+ T cells as measured by production of IFN-γ, IL-2 and TNF-α, did not explain protection across both trials. However, the relative proportion of CD8+ T cell EM vs. CM IFN-γ responses differentiated three of the four protected from the non-protected volunteers across the two trials, especially for AMA1. Non-protected v135 from AdCA was an exception for CSP, exhibiting both a high EM frequency and a high EM:CM ratio.

### CD8+ T cell activities of protected volunteers are directed against single CSP and AMA1 peptide pools

As described above, activities in the DNA/Ad trial, ELISpot and CD8+ T cell IFN-γ and EM activities of protected volunteers appeared to predominantly recognize single CSP or AMA1 peptide pools, whereas non-protected volunteers had lower activities against multiple peptide pools. In addition, as described above, volunteers in the AdCA trial generally recognized multiple peptide pools (Figures S1, S2 and S3). Peptide pool-specificity, while certainly determined by HLA-restricted epitopes that match a volunteer's HLA, these may also reflect patterns of immunodominance that shift during the course of a prime-boost vaccine regimen. Interestingly, one volunteer in the AdCA trial, v194, the only study subject with a significant delay in the onset of parasitemia in that trial (indicating partial protection), also showed a large percent contribution by the dominant peptide pool [26] (Figures S1, S2 and S3). We hypothesize that protection in DNA/Ad trial is mediated by effector T cell activities to specific protective epitopes contained within these predominant peptide pools.

**Figure 7:**
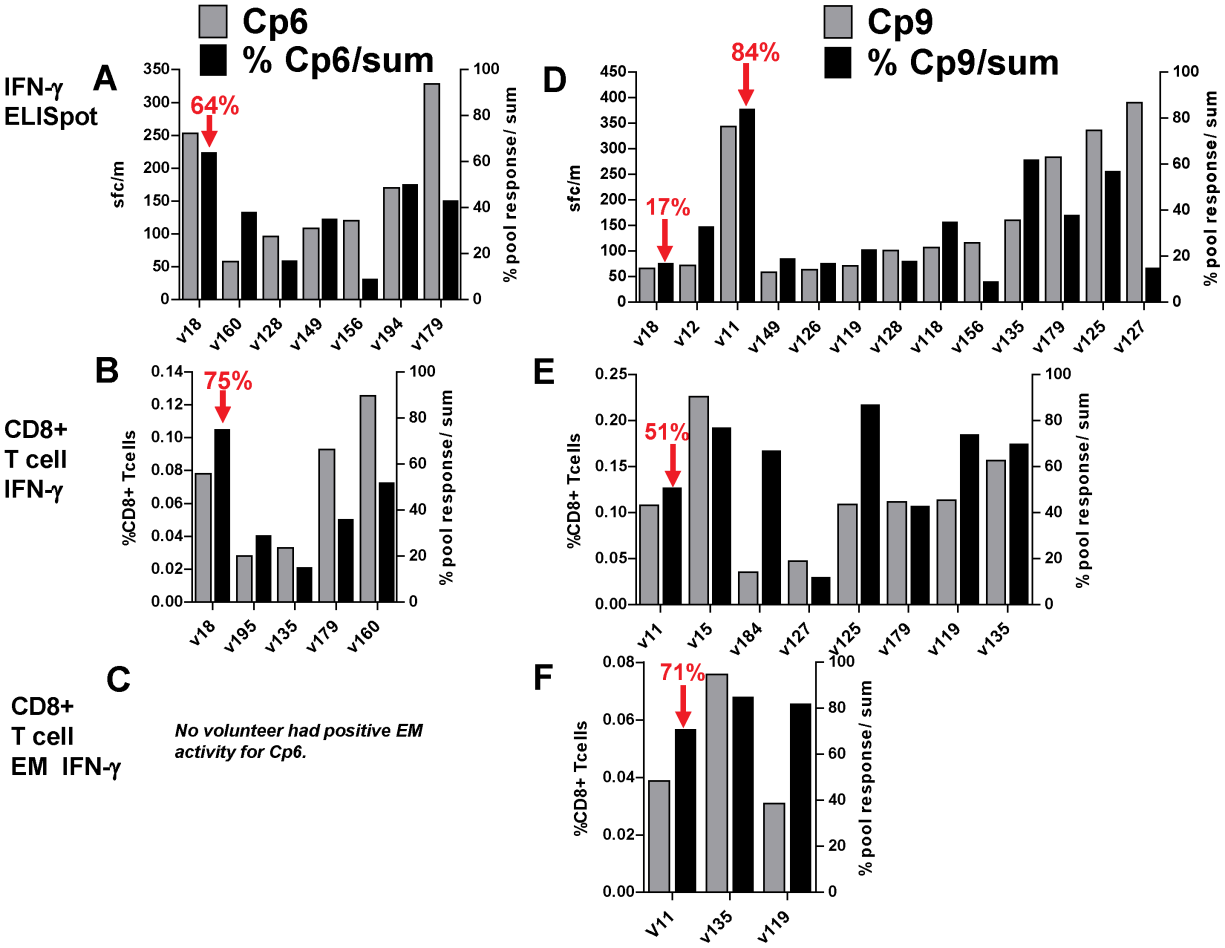
DNA/Ad and AdCA: Comparison of positive T cell activities and percent of total activities to CSP peptide pools. ELISpot, CD8+ T cell IFN-γ, and CD8+ T cell EM IFN-γ activities to individual CSP peptide pools (Cp6 and Cp9) were calculated as per cent of total activities of summed responses to all CSP peptide pools. All volunteers positive with Cp6 or Cp9 were selected. Panels A, B, C: Cp6: protected v18 had lower activities than some AdCA volunteers, but highest per cent activities; v194 (delay to patency) had higher per cent ELISpot activity than other AdCA volunteers, but lower than v18. None of the DNA/Ad or AdCA volunteers had positive EM activity to Cp6. Panels D, E, F: Cp9: protected v11 had highest per cent ELISpot activity to Cp9, but CD8+ T cell IFN-γ and EM activities were lower than some AdCA volunteers.

**Figure 8:**
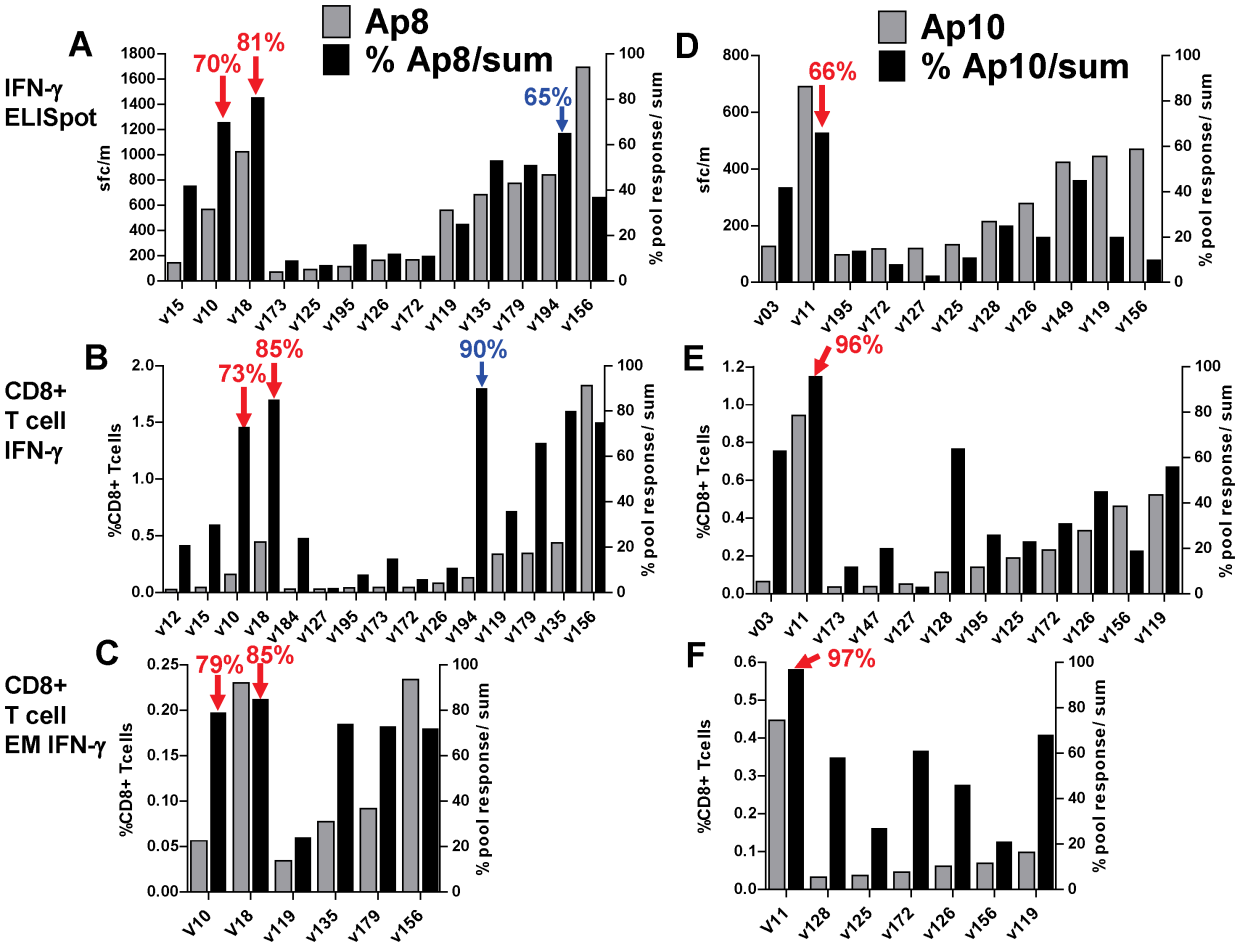
DNA/Ad and AdCA: Comparison of positive T cell activities and per cent of total activities to AMA1 peptide pools. ELISpot IFN-γ, CD8+ T cell IFN-γ, and CD8+ T cell EM IFN-γ activities to individual AMA1 peptide pools (Ap8 and Ap10) were calculated as per cent of total activities of summed responses to all AMA1 peptide pools. All volunteers positive with Ap8 or Ap10 were selected. Panels A, B, C: Ap8: protected v10 and v18 had lower activities than one or more AdCA volunteers, but highest per cent activities; v194 (delay to patency, blue arrows) had higher per cent ELISpot and CD8+ T cell IFN-γ activities than other AdCA volunteers, but no CD8+ T cell IFN-γ EM activity. Panels D, E, F: Ap10: protected v11 had highest per cent ELISpot, CD8+ T cell IFN-γ and CD8+ T cell IFN-γ EM activities to Ap10.

To further examine this apparent focusing of responses to Ad after DNA priming, we compared the percent contribution to the total summed responses of the dominant peptide pool in protected volunteers (Cp6, Cp9, Ap8 and Ap10) to the percent contribution in all non-protected volunteers that were positive to the selected for CSP (Figure 7) and AMA1 (Figure 8) peptide pools. By ELISpot (Figure 7, Panel A; Figure 8, Panel A), the highest percent contributions were: Cp6 in v18, Cp9 in v11, Ap8 in v10 and v18, and Ap10 in v11. Non-protected volunteers from the DNA/Ad and AdCA trials had lower percent activities to specific peptide pools, even though activities to individual peptide pools sometimes exceeded those of protected volunteers, for example the response of v156 response to Ap8. By flow cytometry CD8+ T cell IFN-γ activities (Figure 7, Panel B; Figure 8, Panel B), and CD8+ T cell EM IFN-γ activities (Figure 7, Panel C; Figure 8, Panel C) these relationships obtained with ELISpot were generally maintained, especially for Cp6 and Ap10, noting, however, that for both assays, only immunodominant pools were tested.

The possibility that the focused activities seen in three protected volunteers might be directed to specific class 1-restricted epitopes led us to identify the individual 15-mer peptides within these pools containing the minimal class 1 epitopes that recalled the dominant IFN-γ CD8+ T cell responses in these volunteers.

### Identifying class I-restricted epitopes in CSP and AMA1 predicted to bind to MHC of protected and non-protected volunteers

We used the NetMHC [39] and SYFPEITHI [40] algorithms, which predict peptide/HLA binding affinities, to predict HLA A- and B-restricted epitopes that matched the HLA of the protected volunteers (v10, v11 and v18) and were derived from the peptide pools that were immunodominant in these same volunteers (Cp6, Cp9, Ap8, Ap10). We also predicted epitopes for all other non-protected volunteers with positive activities to these peptide pools. These were v03, v12 and v15 from DNA/Ad (non-protected high responders); non-protected v156 from AdCA (who showed a magnitude of CD8+ T cell activities to Ap8 that was higher than protected v10 and v18); and partially-protected v194 from AdCA (who showed a focused response to Ap8 as well as a significant delay to parasitemia). The epitopes predicted to have strong binding affinities (IC_50_<500 nM) for these volunteers are listed in Table S1.

#### Specific 15-mers recall responses in protected volunteers

To determine whether volunteers recognized the predicted epitopes within the immunodominant pools to which they responded, we first used ELISpot assays to measure responses to each of the individual 15mer peptides comprising the specific pool in question as well as to the total pool. As PBMC after the Ad boost were limited, we also used PBMC collected after challenge, as the pool-specificity of T cell activities was maintained, even though magnitudes of response were reduced after CHMI, perhaps due to localization of circulating antigen-specific T cells into the liver (Figure S6) [26]). Only AMA1 15mer peptides could be tested due to PBMC limitations. Single 15mer peptides were tested with the parent peptide pool in the same assay. All peptide sequences are those of clone CD7 used in the vaccine constructs and CHMI.

#### Ap8 15mers

Protected volunteers v10 and v18 recognized Ap8 15mer peptides A97 and A98 that contained the B*57:01/B*58:01/B*58:02-restricted epitope KSHGKGYNW. The magnitudes of responses to A97 for v10 and v18 were similar to the responses to the entire Ap8 pool. KSHGKGYNW was predicted to be a strong binder for each of these volunteers, with affinities (IC_50_) of 43 nM and 21 nM for v10 and v18, respectively (Table 2 and Table S1). KSHGKGYNW was thus predicted as a B*58 supertype [41]-restricted candidate protective epitope within AMA1. Given the low responses to the other15mer peptides within the Ap8 pool, A93, A94 and A104 by v18, it is unlikely that the three additional epitopes predicted for this volunteer, within these 15mers, each restricted by A02, contributed to protection. No additional high affinity epitopes were predicted for v10 within the thirteen 15mer components of Ap8 (Table 2). Partially protected volunteer v194 was also positive with A97, with the smaller magnitude of response likely reflecting the delayed sampling of PBMC 12 weeks after challenge. V156 was positive with A95 and A97 suggesting that v156 activity may have been directed to A*03:01- and B*58:02-restricted epitopes predicted within these 15mers, SAFLPTGAFK and KSHGKGYNW, respectively (Table 2). However, as this volunteer was not protected, these epitopes may not be protective when recognized through these allele types (even though B*58:02 belongs to the B*58 supertype). Alternatively, responses to these epitopes might be protective but activities were not sufficient to confer protection.

**Table 2:** ELISpot responses to Ap8 and Ap10 peptide pools and individual 15mer peptides within each pool.

Pool	15mer	Sequence	v10^2^	v11^1^	v18^2^	V156^3^	V194^3^
			Prot.	Prot.	Prot.	NP	Delay
Ap8			**193**		**321**	**141**	**47**
	A92	EGFKNKNASMIKSAF	1		0	0	4
	A93	NKNASMIKSAFLPTG	0		2	0	4
	A94	SMIKSAFLPTGAFKA	9		10	11	6
	A95	**SAFLPTGAFK** ADRYK	9		2	**61**	4
	A96	PTGAFKADRYKSHGK	1		2	0	3
	A97	FKADRY**KSHGKGYNW**	**164**		**270**	**141**	**48**
	A98	RY**KSHGKGYNW** GNYN	**96**		**85**	**14**	7
	A99	HGKGYNWGNYNTETQ	0		5	5	6
	A100	YNWGNYNTETQKCEI	1		5	0	6
	A101	NYNTETQKCEIFNVK	0		0	1	0
	A102	ETQKCEIFNVKPTCL	0		0	0	6
	A103	CEIFNVKPTCLINNS	1		0	1	1
	A104	NVKPTCLINNSSYIA	3		0	0	0
**Ap10**				**159**			
	A118	EGNKKIIAPRIFISD		2			
	A119	KIIAPRIFISDDKDS		0			
	A120	PRIFISDDKDSLKCP		1			
	A121	ISDDKDSLKCPCDPE		3			
	A122	KDSLKCPCDPEMVSN		2			
	A123	KCPCDPEMVSNSTCR		1			
	A124	DPEMVSNSTCRFFVC		0			
	A125	VSN**STCRFFVCK** CVE		**140**			
	A126	TCRFFVCKCVERRAE		9			
	A127	FVCKCVERRAEVTSN		4			
	A128	CVERRAEVTSNNEVV		10			
	A129	RAEVTSNNEVVVKEE		0			
	A130	TSNNEVVVKEEYKDE		0			

1 DNA/Ad cells collected 28 days after malaria challenge;

2 DNA/Ad cells collected 84 days after malaria challenge.

3 AdCA cells collected 2 months after the malaria challenge. Prot.  =  Protected. NP = Not Protected. All 15mer peptides within Ap8 and Ap10 were tested with DNA/Ad, and AdCA-immunized volunteers in ELISpot assays. Positive activities (bold): activity was greater than twice that of control, and the difference in absolute count was >10 sfc/m. Predicted epitopes within positive 15mers are shown in bold and underlined.

#### Ap10 15mers

Only protected v11 was tested, and recognized the Ap10 15mer peptide A125 containing the predicted A*11:01-restricted STCRFFVCK (A03 supertype [41]) that was predicted to be a very strongly binding epitope for this volunteer, with an IC50 value of 7 nM. The magnitude of the response to A125 was similar to the magnitude of the response to the entire Ap10 pool, and therefore this epitope was also identified as a candidate protective epitope within AMA1. Several other epitopes predicted for v11 within Ap10, also A03-restricted, were likely non-contributory as there were no or minimal responses to the corresponding 15mers.

#### HLA-restricted class I AMA1 epitopes recall responses in protected volunteers

Two candidate protective class 1-restricted AMA1 epitopes KSHGKGYNW and STCRFFVCK were synthesized and tested with the parent peptide pools for recall of T cell activities (Table 3). Since frozen PBMC were used, ELISpot activities in particular with the parent peptide pools were lower than with fresh PBMC (Figure 2, Panel C).

**Table 3:** ELISpot and flow cytometry activity of synthesized AMA1 epitopes with protected volunteers.

Vol.	Status	Vol. HLA	Peptide Pool	Epitope	HLA-restr.	ELISpot sfc/m	% CD8+ T Cell IFN-γ	% CD8+ T Cell EM IFN-γ
**v10**	Protected	B*57:01	Ap8			131^2^	0.06^1^	0.03^1^
				YKSHGKGYNW	B*57:01	159^2^	0.05^1^	0.025^1^
**v18**	Protected	B*58:01	Ap8			158^2^	0.64^1^	0.21^1^
				YKSHGKGYNW	B*58:01	181^2^	0.86^1^	0.31^1^
**v156**	Non-protected	B*58:02	Ap8			141^4^	1.11^B2^	0.25^3^
				KSHGKGYNW	B*58:02	88^4^	1.47^B2^	0.40^3^
**v194**	Partially-protected	B*58:01	Ap8			47^4^	0.17^B2^	0.07^3^
				KSHGKGYNW	B*58:01	19^4^	0.22^B2^	0.08^3^
**v11**	Protected	A*11:01	Ap10			NT	0.37^1^	0.10^1^
				NSTCRFFVCK	A*11:01	248^1^ 231^1^	0.39^1^	0.14^1^

1 DNA/Ad cells collected 22/23 days after Ad boost.

2 DNA/Ad cells collected 84 days after malaria challenge.

3 AdCA cells collected 22/23 days after AdCA immunization.

4 Cells collected 84 days after malaria challenge. Each synthesized AMA1 predicted epitope was tested in ELISpot and flow cytometry with the parent Ap8 or Ap10 peptide pool. NT = Not Tested. Volunteer v11 was tested twice in ELISpot with predicted epitope. The Ap8 peptide pool and corresponding KSHGKGYNW (or contained within the 10mer YKSHGKGYNW) epitope recalled similar ELISpot from v10 and v18, but weaker CD8+ T cell activities from v10 than v18 (consistent with Figures 2 and 3). Ap8 and KSHGKGYNW recalled activities from partially protected v194 (consistent with Figure S1, S2, but not Figure S3 where v156 lacked EM activity). Ap8 and KSHGKGYNW also recalled activities from non-protected v156 (consistent with Figures S1, S2 and S3). Ap10 and the corresponding epitope STCRFFVCK (contained within the 10mer NSTCRFFVCK) recalled activities from v11 (consistent with Figures 2, 3 and 4).

The AMA1 B*57:01/B*58:01-restricted epitope KSHGKGYNW, and the parent peptide pool Ap8, were positive in ELISpot, CD8+ T cell IFN-γ and CD8+ T cell EM IFN-γ assays with protected volunteers v10 and v18, and partially protected v194 (although not in ELISpot, probably as post-challenge PBMC were used), as predicted from 15mer results. This epitope also recalled robust activities from non-protected v156 that may be B*58:02-restricted, with the difference in HLA restriction perhaps explaining why v156 was not protected. The AMA1 A*11:01-restricted epitope STCRFFVCK, contained within NSTCRFFVCK, and the parent peptide pool Ap10, were also positive in ELISpot, CD8+ T cell IFN-γ and CD8+ T cell EM IFN-γ activities with protected v11 as predicted. For all three protected volunteers studied – v10, v11 and v18 – responses recalled by the minimal epitope were as strong as those recalled by the parent peptide pool (Table 3).

In summary, our preliminary mapping studies indicated that specific minimal epitopes predicted to bind to the HLA of protected v10, v11, and v18 may have been responsible for the pool-specific activity in these volunteers. We have therefore identified KSHGKGYNW and STCRFFVCK from AMA1 as potentially important epitopes underlying the protection seen in the DNA/Ad trial. However, non-protected volunteers such as v156, a different allele type (B*58:02) but belonging to the same superfamily (B*58), shared recognition, indicating that other factors, such as the specific HLA allele and the quality of the responding T cell populations, were also important.

## Discussion

The first clinical trials of DNA prime/viral vector boost malaria vaccines used pox-vectors such as MVA for boosting and induced only limited protection in humans [10], [18], [42]. Our DNA/Ad clinical trial was the first to use adenovectors for boosting after a DNA prime (NMRC-M3V-D/Ad-PfCA vaccine). This approach, selected due to the superior ability of adenovectors to induce CD8+ T cell responses in humans [36], [43], induced the highest level of sterile protection against malaria (27%) seen to date in a clinical trial using gene-based vaccine platforms [19]. Immunity was significantly associated with IFN-γ-producing CD8+ T cells, a first for a malaria subunit vaccine in humans [19], confirming the finding in animal models that these effectors can mediate protection [10], [44], [45]. Subsequent work using an adenovirus prime/MVA boost regimen has confirmed the association of CD8+ T cells and protection against the pre-erythrocytic stages of malaria in humans [20].

The aim of our study was to more fully explore the immune activities in the DNA/Ad trial that were associated with protection. Since the Ad boost vaccine (NMRC-M3V-Ad-PfCA), administered alone in a separate study (AdCA trial), was strongly immunogenic but did not elicit sterile protection [26], we were able to broaden our investigation by combining samples from the two clinical studies. This allowed an examination of the effect of DNA-priming on Ad-induced responses and how this may have enhanced protection.

The findings of our exploratory investigation were complex and unexpected. DNA priming has previously been shown to increase CD8+ T cell responses in animal models [46] and in humans [47], even in the absence of detectable post-DNA activities [47], [48]. However, we found that, overall, DNA priming appeared to affect summed CD8+ T cell IFN-γ activities following Ad, and this was most apparent when the activities of non-protected volunteers in DNA/Ad were compared to those of non-protected volunteers receiving the Ad vaccine alone. Although ELISpot and CD8+ T cell IFN-γ activities were both significantly higher in protected than non-protected volunteers within the DNA/Ad trial, they were lower than those found in many of the non-protected volunteers in AdCA, indicating that these peripheral responses qualified as correlates of protective immunity only in the DNA/Ad trial. We recognize that absolute numbers of these T cell populations may be important, and that extremely high frequencies of memory CD8+ T cells may be required for long-term protection, at least in mice [49]. This prompted a more detailed investigation into the nature of T cell responses in the two trials. We hoped to identify correlates bridging across both studies, while recognizing that correlates might ultimately prove to be different following DNA/Ad and Ad alone.

We found that DNA priming influenced the relative magnitudes of memory populations, increasing EM relative to CM activities in some protected volunteers relative to non-protected volunteers in both trials. Without DNA priming, EM responses, while robust in most non-protected volunteers, were overshadowed by CM responses, reducing the EM/CM ratios. It is possible that this may reflect T cell exhaustion [27], [38], [50], [51] as previously suggested for Ad5 [52]. We are planning to examine the potential for T cell exhaustion in future trials by monitoring the expression of PD-1 and other associated markers [52], [53].

Protection in mice against malaria is associated with memory T cell responses [7] and malaria vaccines induced memory responses in humans [18], [54]–[56]. This was evident in the DNA/Ad trial that induced EM activities in protected but non-protected volunteers, although EM activities in the AdCA trial were higher in some of these volunteers. However, the higher proportion of EM to CM activities to AMA1 (and possibly CSP) distinguished some protected volunteers from the non-protected volunteers in both trials. This confirms an earlier study in mice [57], where a protective prime-boost regimen induced higher EM than CM activities whereas a less protective regimen induced higher CM than EM activities. In addition to affecting the relative proportions of EM and CM activities in protected volunteers, DNA-priming appeared to focus T cell activities to discrete regions of CSP and/or AMA1 represented by single peptide pools, when compared to the broader specificities induced by AdCA immunization. We propose that protection after DNA/Ad immunization requires that T cell activities, especially effector memory responses, reach a threshold in magnitude and target a specific peptide pool presumed to contain protective epitopes. Volunteers that were not protected lacked this focus on specific areas of the vaccine antigens, or lacked a sufficient magnitude of response.

While the focused CD8+ T cell responses in three of the protected volunteers (v10, v11 and v18) contrasted with the broad responses in non-protected volunteers, they were not evident in protected v06, who had low T cell activities to multiple pools and no measurable memory responses. In this volunteer, antigen-specific CD8+ T cells may have been almost completely localized to the liver and therefore may have been difficult to detect in the periphery [28], [29]. In this context, the finding that malaria-induced liver resident CD8+ T cells display a transcriptional profile that differs for those described for other microbial challenges could be an important step to determining liver-resident CD8+ T cells in humans [58]. It is also possible that CD4+ T cells or non-lymphocyte immune cells may have mediated protection.

The mechanisms by which DNA priming affected the strong T cell responses after the Ad boost, favoring the induction of EM over CM responses and promoting recognition of discrete regions of the vaccine antigens, are not clear. It is possible that effector T cell populations targeting malaria antigens induced by DNA priming reduced the level and duration of transgene expression and antigen presentation following Ad boost [59], [60] or these effector populations may have differentially killed APCs or other cells presenting epitopes in response to Ad that had been immunodominant after the DNA prime [59], [60], allowing T cells with specificities recognizing more protective epitopes to be differentially stimulated by Ad. An extensive examination of epitope specific responses to these two vaccine regimens after DNA and after Ad could address this hypothesis in future studies.

Although PBMC were limited, we were able to map and confirm recognition of the B*57:01/B*58:01-restricted epitope KSHGKGYNW and the A*11:01-restricted epitope STCRFFVCK, which recalled CD8+ T cell IFN-γ and EM responses from v10, v18, and v11 as predicted. The potential association between responses to these epitopes and protection was supported by the recall activities of the B*58:01-restricted epitope KSHGKGYNW by partially protected v194 from the AdCA trial. KSHGKGYNW is variable at position 393 (H or R) and position 395 (K or R) and STCRFFVCK is variable at position 503 (R or N) and position 505 (F or Y) [61], [62]. However, NetMHC predicted similar binding affinities to B*58:01 and A*11:01, respectively (data not shown), suggesting that these variant epitope sequences may elicit similar ELISpot and CD8+ T cell activities. To our knowledge, the vaccine tested in the DNA/Ad trial (NMRC-M3V-D/Ad-PfCA) is the first malaria vaccine to induce protection in humans that has been linked to specific class 1-restricted epitopes. Because protection was not induced in one volunteer from AdCA expressing HLA B*58:02 (also B58 supertype), we wondered if the specific HLA type might be an important determinant of protection. Heterozygous B*27, B*57 and B*58 are significantly associated with slower progress from HIV infection to illness and with lower mortality, resulting in long term non-progression. Recently, T regulatory cells (Tregs) have been shown to suppress proliferation of HIV-specific CD8+ cytotoxic T cells (CTLs) during chronic infection but B*57-restricted cells evaded suppression by killing Tregs [63]. If this finding also applies to malaria, it is possible that DNA priming induced Tregs that suppressed Ad-induced CD8+ T cell IFN-γ responses in the majority of volunteers expressing HLA alleles other than B*57 and B*58, and that these two HLA alleles offered a degree of resistance to the effects of Tregs. In future studies, we therefore plan to investigate the role of Tregs to selectively suppress responses in volunteers with different HLA allele groups.

Our study indicated that IFN-γ monofunctional CD8+ T cells in three volunteers and IFN-γ/TNF-α polyfunctional CD8+ T cells in one volunteer constituted the primary subpopulations of CD8+ T cell responses in protected volunteers, although no correlates of protection could be identified examining differences in cytokine profiles. Studies in mice have also suggested that protection was associated with IFN-γ monofunctional and IFN-γ/TNF-α polyfunctional activities [37]. As the DNA/Ad regimen is repeated in the future, the role of mono vs. polyfunctional activities will be examined in greater detail.

## Conclusions

This is the first malaria vaccine tested in humans shown to induce protection associated with CD8+ T cell activities. Here we show that in three of four protected volunteers, these activities involved the recognition of specific class 1-restricted AMA1 epitopes. Future research will endeavor to establish conditions, such as better-designed vaccine antigens and immunization regimens that reproducibly elicit protective responses in a larger proportion of volunteers. The use of rare serotype adenovectors may modulate the strong inflammatory component of Ad5 and also avoid pre-existing immunity, therefore serving as more effective boosts in heterologous regimens. The findings of this study should be broadly applicable to other pathogens where CD8+ T cell responses may contribute to protection.

## Supporting Information

Figure S1 **AdCA:** ***Ex vivo*** **T cell IFN-γ activities by ELISpot Assay to CSP and AMA1.** ELISpot activities against CSP and AMA1 peptide pools are shown as color-coded bars at pre-immunization (1) and 22–23 days after Ad immunization. Since no volunteer was protected volunteers are grouped numerically. Boxed volunteer (v194) was partially protected. *Positive activities after Ad immunization. Panel A: CSP: 14/18 volunteers were positive; Panel B: AMA1: 18/18 volunteers were positive.
(TIFF)

Figure S2 **AdCA: CD8+ T cell IFN-γ activities to CSP and AMA1.** CD8+ total IFN-γ against CSP peptide pools are shown as color-coded bars at pre-immunization (1) and 22–23 days after Ad immunization (2). Volunteers are grouped numerically. Boxed volunteer (v194) was partially protected. *Positive activities after Ad immunization. Panel A: CSP: 12/16 volunteers were positive. Panel B: AMA1: 16/16 volunteers were positive.
(TIFF)

Figure S3 **AdCA CD8+ NV, CM, EM and TD T cell IFN-γ activities to CSP and AMA1.** CD8+ Memory naïve (N), central (C), effector (E) and terminally differentiated (T) T cells are shown as per cent of CD8+ T cells to CSP and AMA1 peptide as color-coded bars. Volunteers are grouped numerically. Boxed volunteer (v194) was partially protected. *Positive activities after Ad immunization. CSP: NV, CM, EM and TD activities were positive with 2/16, 8/16, 3/16 and 1/16 volunteers. AMA1: NV, CM, EM and TD activities were positive with 6/16, 15/16, 10/16 and 7/16 volunteers. Geometric means of CM and EM activities to AMA1 (0.26%, 0.07%) were higher than those of CSP (0.05%, <0.03%).
(TIFF)

Figure S4 **DNA/Ad and AdCA CD8+ T cell monofunctional and polyfunctional memory activities to CSP.** Monofunctional and polyfunctional CD8+ T cell memory (CM, EM and TD) activities to CSP after DNA/Ad (Panel A) or AdCA immunization (Panel B) are shown as color-coded filled circles that represent the per cent of CD8+ T cells containing cytokine(s) as indicated. Panel A: DNA/Ad: CSP: Only protected v11 (0.06%) and v18 (0.05%), and non-protected v15 (0.5%) developed IFN CM monofunctional activities; v15 also developed high CM IL2 monofunctional activity (0.19%); however, (in agreement with Figure 2), IFN monofunctional EM activities only developed in protected v11 (0.04%) and v18 (0.04%) and were negative on v15; however, v15 developed low EM IL2 monofunctional (0.05%) activity. No protected volunteers had TD activities (in agreement with Figure 2). Panel B: AdCA: CSP: 7/16 volunteers developed IFN monofunctional CM activities of whom six were higher than protected volunteers (and excluding v15); two/16 volunteers IL2 monofunctional CM activities absent in protected volunteers; three/16 volunteers developed only IFN monofunctional activities that were higher than protected volunteers; one/16 volunteers developed only IFN monofunctional activity that was absent in protected volunteers.
(TIFF)

Figure S5 **DNA/Ad and AdCA CD8+ T cell monofunctional and polyfunctional memory activities to AMA1.** Monofunctional and polyfunctional CD8+ T cell memory (CM, EM and TD) activities to AMA1 after DNA/Ad (Panel A) or AdCA immunization (Panel B) are shown as color-coded filled circles that represent the per cent of CD8+ T cells containing cytokine(s) as indicated. Arrows indicate summed activities that exceed the positive cut off but were considered as negative as activities to individual peptide pools were all negative. Panel A: DNA/Ad: AMA1: three protected volunteers developed positive IFN monofunctional CM (v10: 0.06%; v11: 0.14%; v18: 0.16%), EM (v10: 0.05%; v11: 0.27%; v18: 0.25%), and TD activities that were positive on two protected volunteers (v11: 0.10%; v18: 0.07%). In addition, v11 also developed IFN/TNF polyfunctional CM (0.05%), EM (0.15%) and TD (0.06%) that represented 34%, 56% and 63% of total IFN activity. Only one non-protected volunteer developed IFN monofunctional CM activity (0.11%) but not EM or TD activities. Three non-protected volunteers developed summed CM, and two non-protected volunteers developed EM, activities that were similar to v10, but were not considered positive as activities to individual AMA1 peptide pools were negative. Panel B: AdCA: AMA1: 15/16 volunteers developed positive IFN monofunctional CM activities (range 0.08%–1.1%) and of these 12 were higher than the protected volunteers; in addition three volunteers developed IFN/IL2 polyfunctional or IL2 monofunctional CM activities that were absent in protected volunteers. Ten/16 volunteers developed IFN monofunctional EM activities (0.05–0.28%) and of these only one was higher than two of the protected volunteers, v18 and v11, but all were higher than protected v10. Four/16 volunteers developed positive TD IFN monofunctional activities and three of these were higher than protected v11 and v18.
(TIFF)

Figure S6 **DNA/Ad: Pre- and post-challenge T cell responses to CSP and AMA1 peptide pools.** The T-cell activities of protected volunteers v06, v10, v11 and v18 to CSP and AMA1 peptide pools were measured 22/23 days after the Ad boost, 5/6 days before malaria challenge (1), 28 days (2) and 84 days (3) after malaria challenge. Panels A and B: ELISpot activities; Panels C and D: CD8+ T cell EM IFN-γ activities. With ELISpot and CD8+ T cell EM IFN-γ activities at 28 days after challenge of protected volunteer v11 fell with Cp9, v11 fell with Cp6, v10 and v18 fell with Ap8 and v11 fell with Ap10, and all activities generally declined further by 84 days after challenge. In contrast ELISpot activities of non-protected volunteers all rose at 28 days after challenge, and then declined by 84 days after challenge (not shown); CD8+ T cell EM IFN-γ activities remained negative after challenge. Therefore, after challenge ELISpot pool-specific activities and CD8+ T cell EM IFN-γ activities, although lower, maintained the same specificity as before challenge, with the exception of v11 CD8+ T cell EM IFN-γ activity with Cp9 that fell below the positive cut off.
(TIFF)

Table S1 **Predicted class 1-restricted epitopes within CSP and AMA1 peptide pools predominantly recognized by protected and non-protected volunteers.**
(DOCX)
