# Supplementary figures and images for: Sterile Immunity to Malaria after DNA Prime/Adenovirus Boost Immunization Is Associated with Effector Memory CD8+T Cells Targeting AMA1 Class I Epitopes
Source: PLoS One. 2014 Sep 11;9(9):e106241. doi: 10.1371/journal.pone.0106241 (PMC4161338; doi:10.1371/journal.pone.0106241)

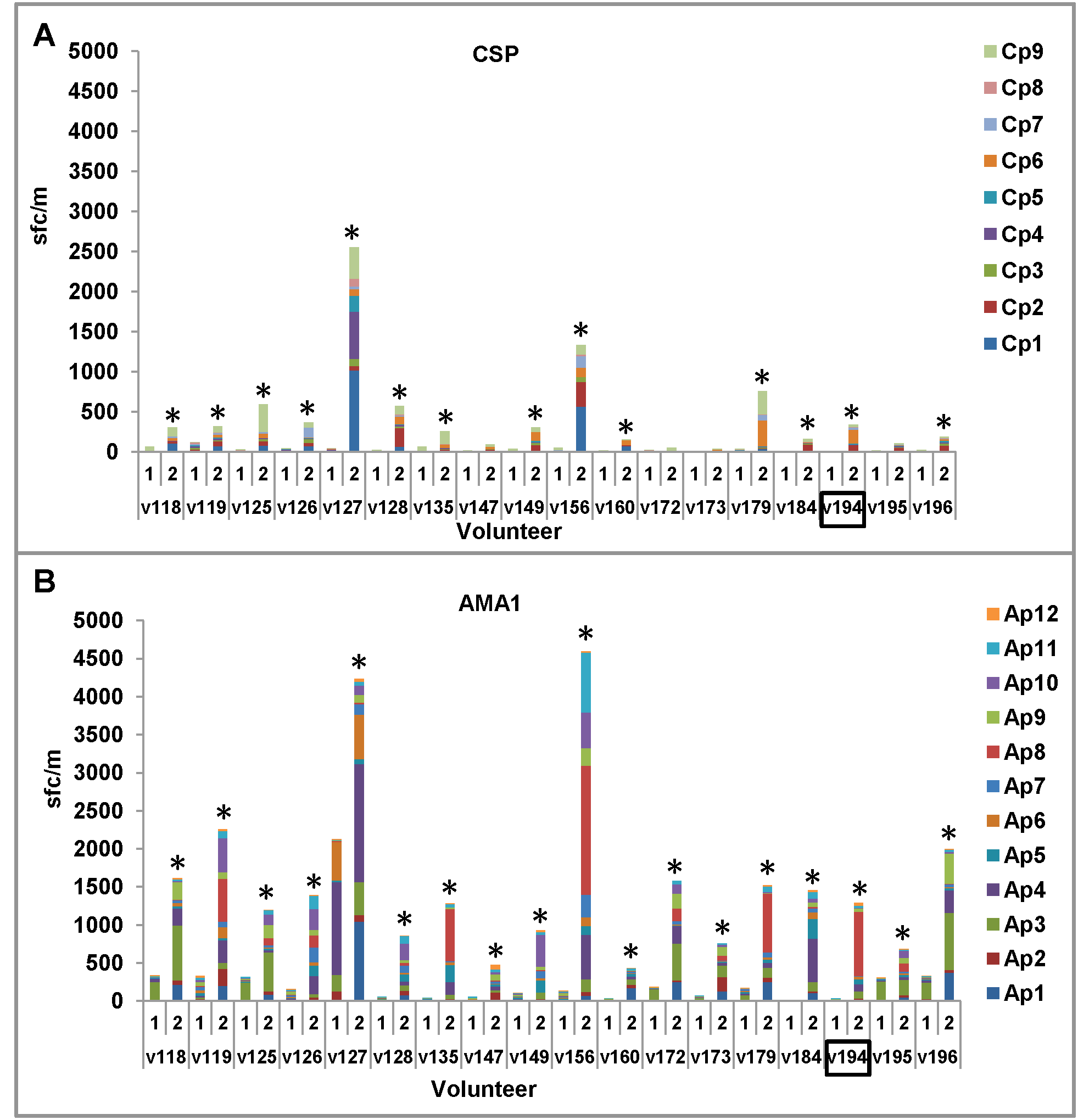

Supplement: Figure S1 — AdCA: Ex vivo T cell IFN-γ activities by ELISpot Assay to CSP and AMA1. ELISpot activities against CSP and AMA1 peptide pools are shown as color-coded bars at pre-immunization (1) and 22–23 days after Ad immunization. Since no volunteer was protected volunteers are grouped numerically. Boxed volunteer (v194) was partially protected. *Positive activities after Ad immunization. Panel A: CSP: 14/18 volunteers were positive; Panel B: AMA1: 18/18 volunteers were positive. (TIFF) [file pone.0106241.s001.tiff]

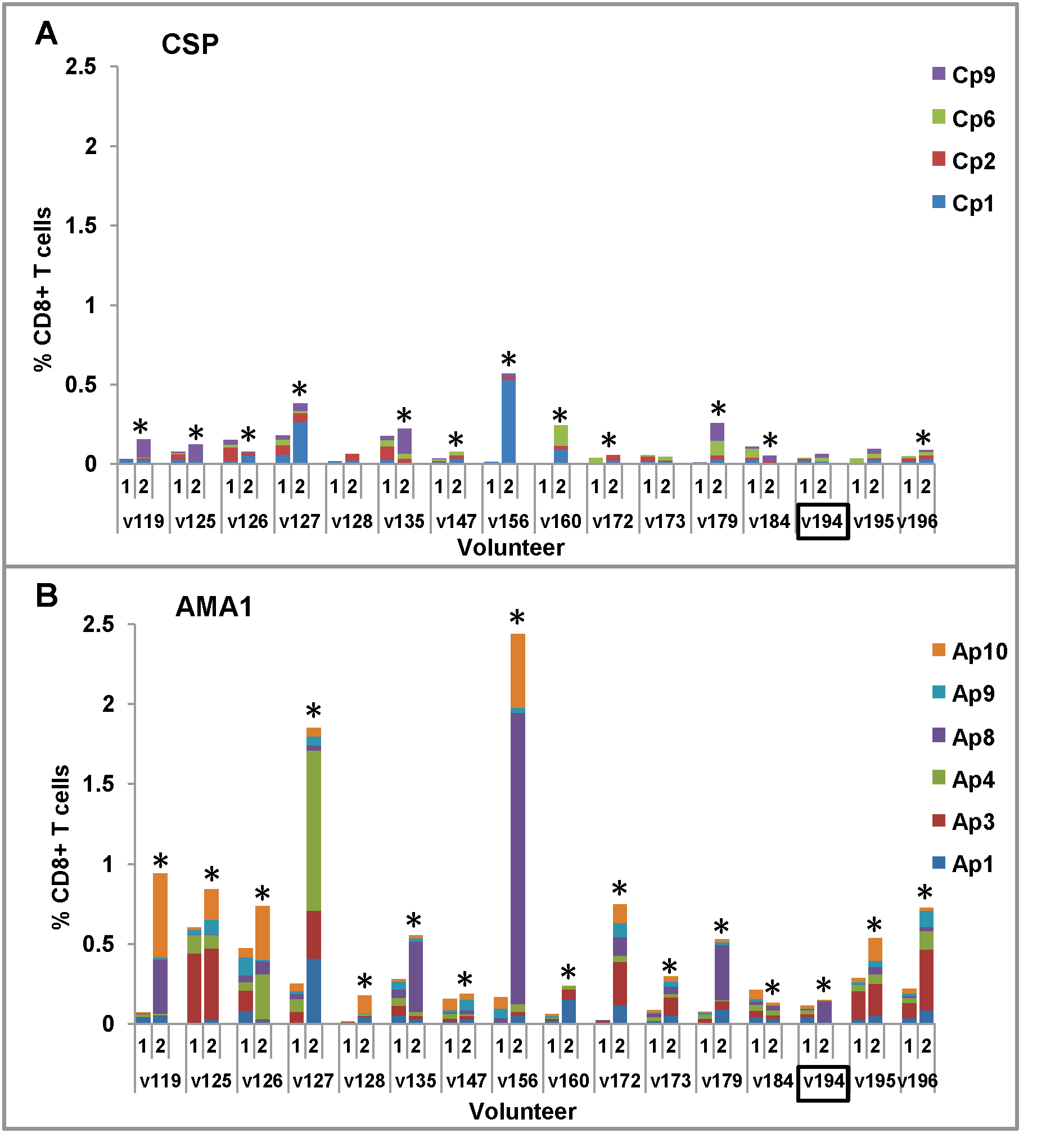

Supplement: Figure S2 — AdCA: CD8+ T cell IFN-γ activities to CSP and AMA1. CD8+ total IFN-γ against CSP peptide pools are shown as color-coded bars at pre-immunization (1) and 22–23 days after Ad immunization (2). Volunteers are grouped numerically. Boxed volunteer (v194) was partially protected. *Positive activities after Ad immunization. Panel A: CSP: 12/16 volunteers were positive. Panel B: AMA1: 16/16 volunteers were positive. (TIFF) [file pone.0106241.s002.tiff]

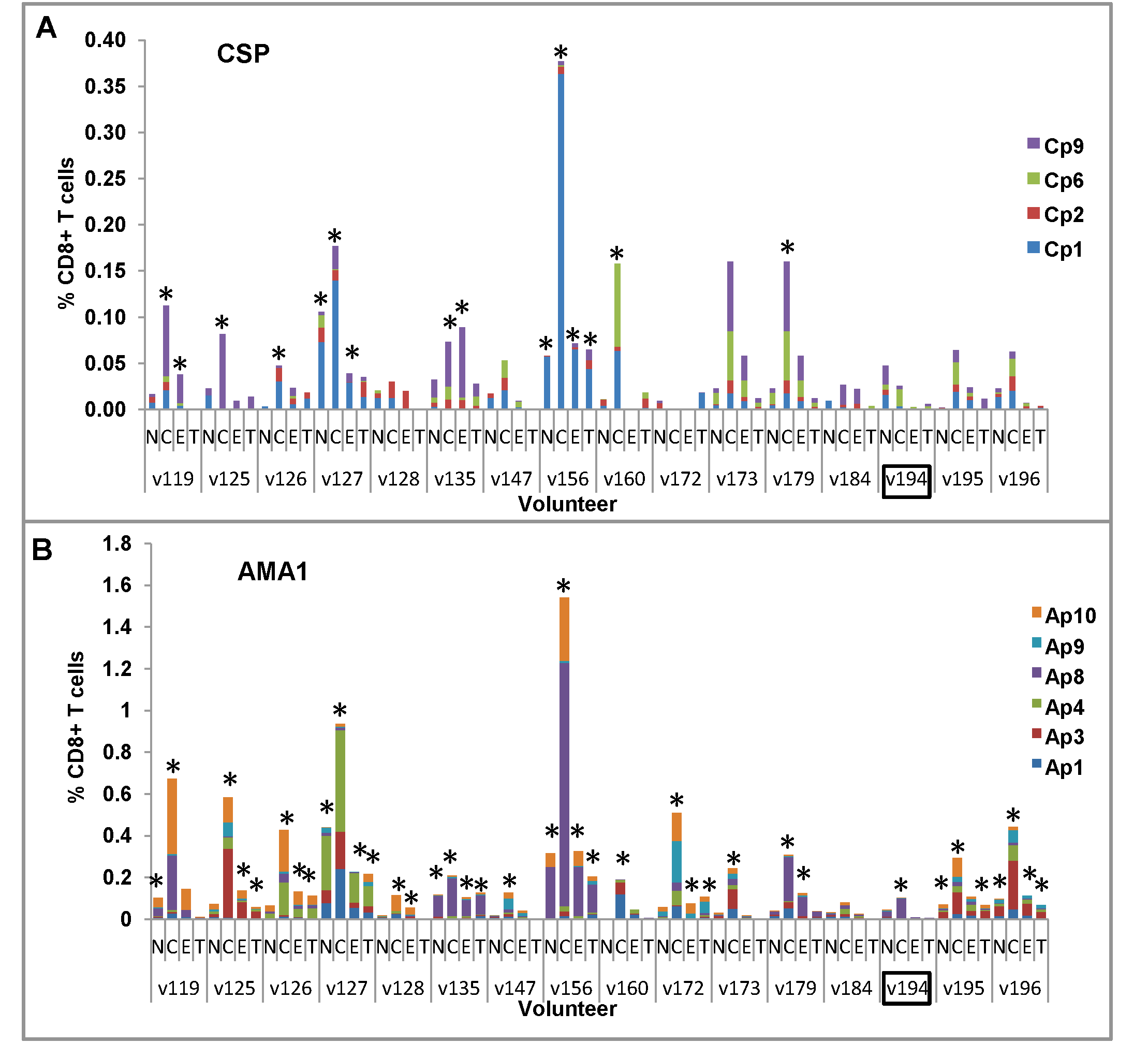

Supplement: Figure S3 — AdCA CD8+ NV, CM, EM and TD T cell IFN-γ activities to CSP and AMA1. CD8+ Memory naïve (N), central (C), effector (E) and terminally differentiated (T) T cells are shown as per cent of CD8+ T cells to CSP and AMA1 peptide as color-coded bars. Volunteers are grouped numerically. Boxed volunteer (v194) was partially protected. *Positive activities after Ad immunization. CSP: NV, CM, EM and TD activities were positive with 2/16, 8/16, 3/16 and 1/16 volunteers. AMA1: NV, CM, EM and TD activities were positive with 6/16, 15/16, 10/16 and 7/16 volunteers. Geometric means of CM and EM activities to AMA1 (0.26%, 0.07%) were higher than those of CSP (0.05%, <0.03%). (TIFF) [file pone.0106241.s003.tiff]

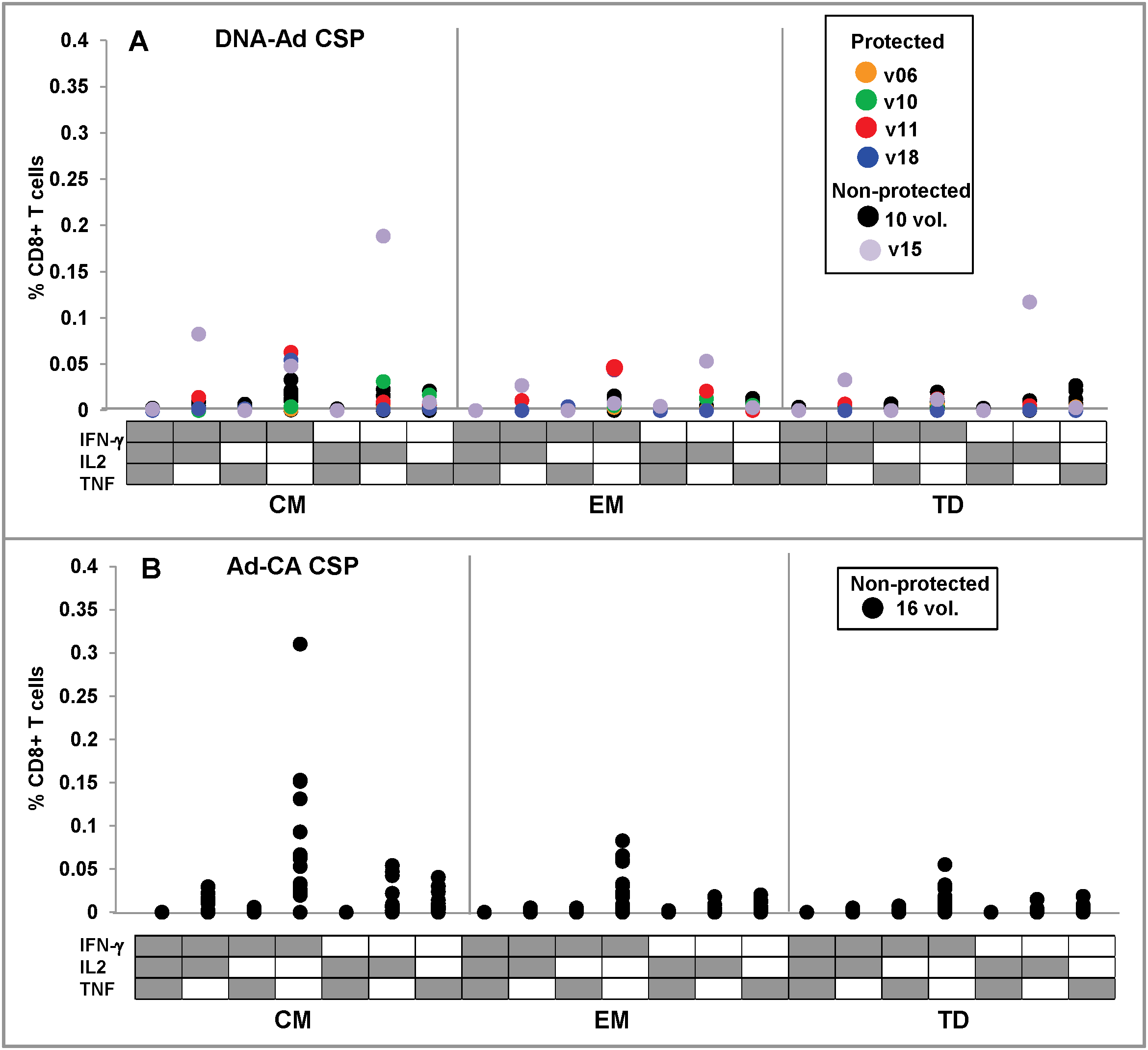

Supplement: Figure S4 — DNA/Ad and AdCA CD8+ T cell monofunctional and polyfunctional memory activities to CSP. Monofunctional and polyfunctional CD8+ T cell memory (CM, EM and TD) activities to CSP after DNA/Ad (Panel A) or AdCA immunization (Panel B) are shown as color-coded filled circles that represent the per cent of CD8+ T cells containing cytokine(s) as indicated. Panel A: DNA/Ad: CSP: Only protected v11 (0.06%) and v18 (0.05%), and non-protected v15 (0.5%) developed IFN CM monofunctional activities; v15 also developed high CM IL2 monofunctional activity (0.19%); however, (in agreement with Figure 2), IFN monofunctional EM activities only developed in protected v11 (0.04%) and v18 (0.04%) and were negative on v15; however, v15 developed low EM IL2 monofunctional (0.05%) activity. No protected volunteers had TD activities (in agreement with Figure 2). Panel B: AdCA: CSP: 7/16 volunteers developed IFN monofunctional CM activities of whom six were higher than protected volunteers (and excluding v15); two/16 volunteers IL2 monofunctional CM activities absent in protected volunteers; three/16 volunteers developed only IFN monofunctional activities that were higher than protected volunteers; one/16 volunteers developed only IFN monofunctional activity that was absent in protected volunteers. (TIFF) [file pone.0106241.s004.tiff]

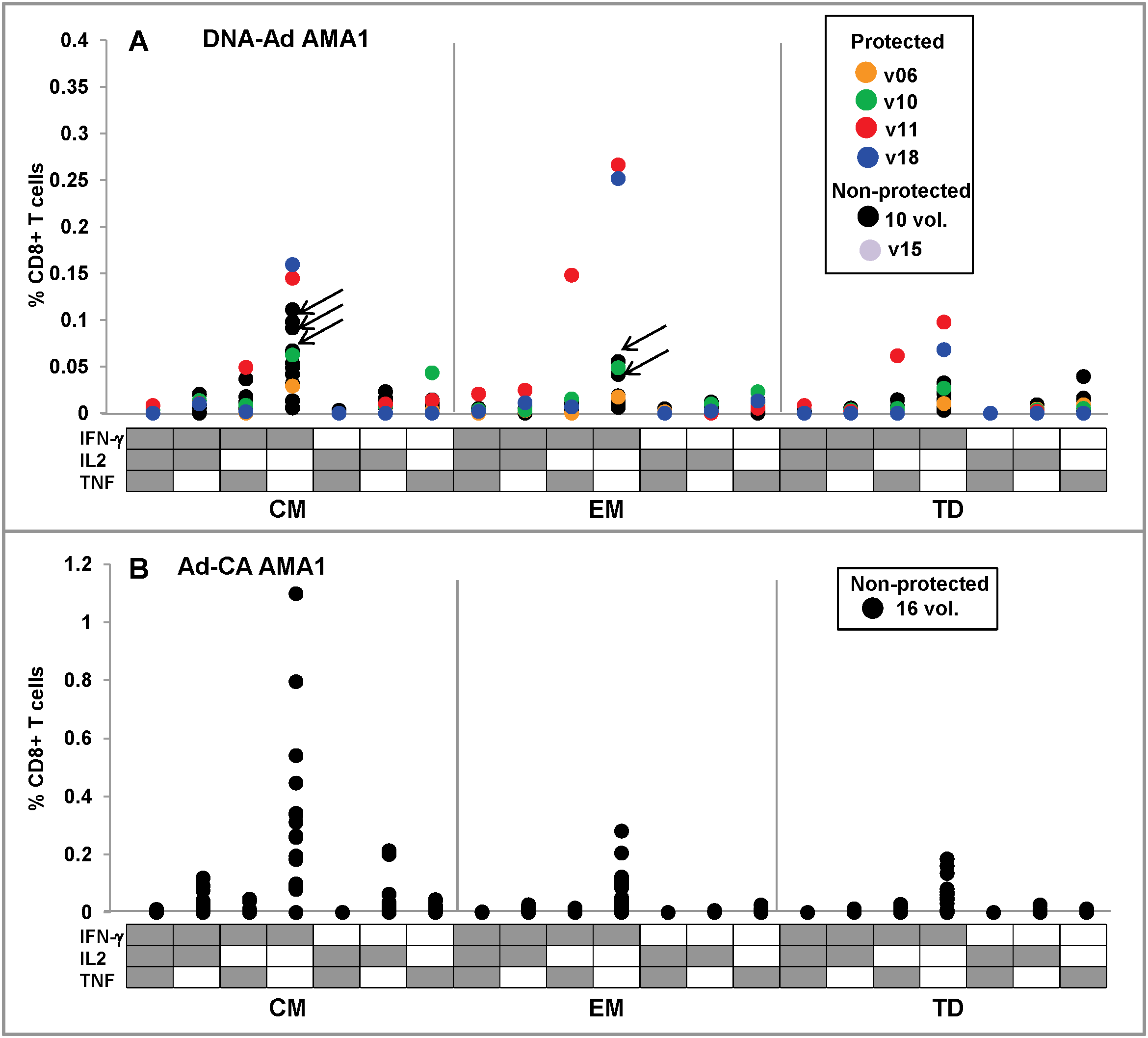

Supplement: Figure S5 — DNA/Ad and AdCA CD8+ T cell monofunctional and polyfunctional memory activities to AMA1. Monofunctional and polyfunctional CD8+ T cell memory (CM, EM and TD) activities to AMA1 after DNA/Ad (Panel A) or AdCA immunization (Panel B) are shown as color-coded filled circles that represent the per cent of CD8+ T cells containing cytokine(s) as indicated. Arrows indicate summed activities that exceed the positive cut off but were considered as negative as activities to individual peptide pools were all negative. Panel A: DNA/Ad: AMA1: three protected volunteers developed positive IFN monofunctional CM (v10: 0.06%; v11: 0.14%; v18: 0.16%), EM (v10: 0.05%; v11: 0.27%; v18: 0.25%), and TD activities that were positive on two protected volunteers (v11: 0.10%; v18: 0.07%). In addition, v11 also developed IFN/TNF polyfunctional CM (0.05%), EM (0.15%) and TD (0.06%) that represented 34%, 56% and 63% of total IFN activity. Only one non-protected volunteer developed IFN monofunctional CM activity (0.11%) but not EM or TD activities. Three non-protected volunteers developed summed CM, and two non-protected volunteers developed EM, activities that were similar to v10, but were not considered positive as activities to individual AMA1 peptide pools were negative. Panel B: AdCA: AMA1: 15/16 volunteers developed positive IFN monofunctional CM activities (range 0.08%–1.1%) and of these 12 were higher than the protected volunteers; in addition three volunteers developed IFN/IL2 polyfunctional or IL2 monofunctional CM activities that were absent in protected volunteers. Ten/16 volunteers developed IFN monofunctional EM activities (0.05–0.28%) and of these only one was higher than two of the protected volunteers, v18 and v11, but all were higher than protected v10. Four/16 volunteers developed positive TD IFN monofunctional activities and three of these were higher than protected v11 and v18. (TIFF) [file pone.0106241.s005.tiff]

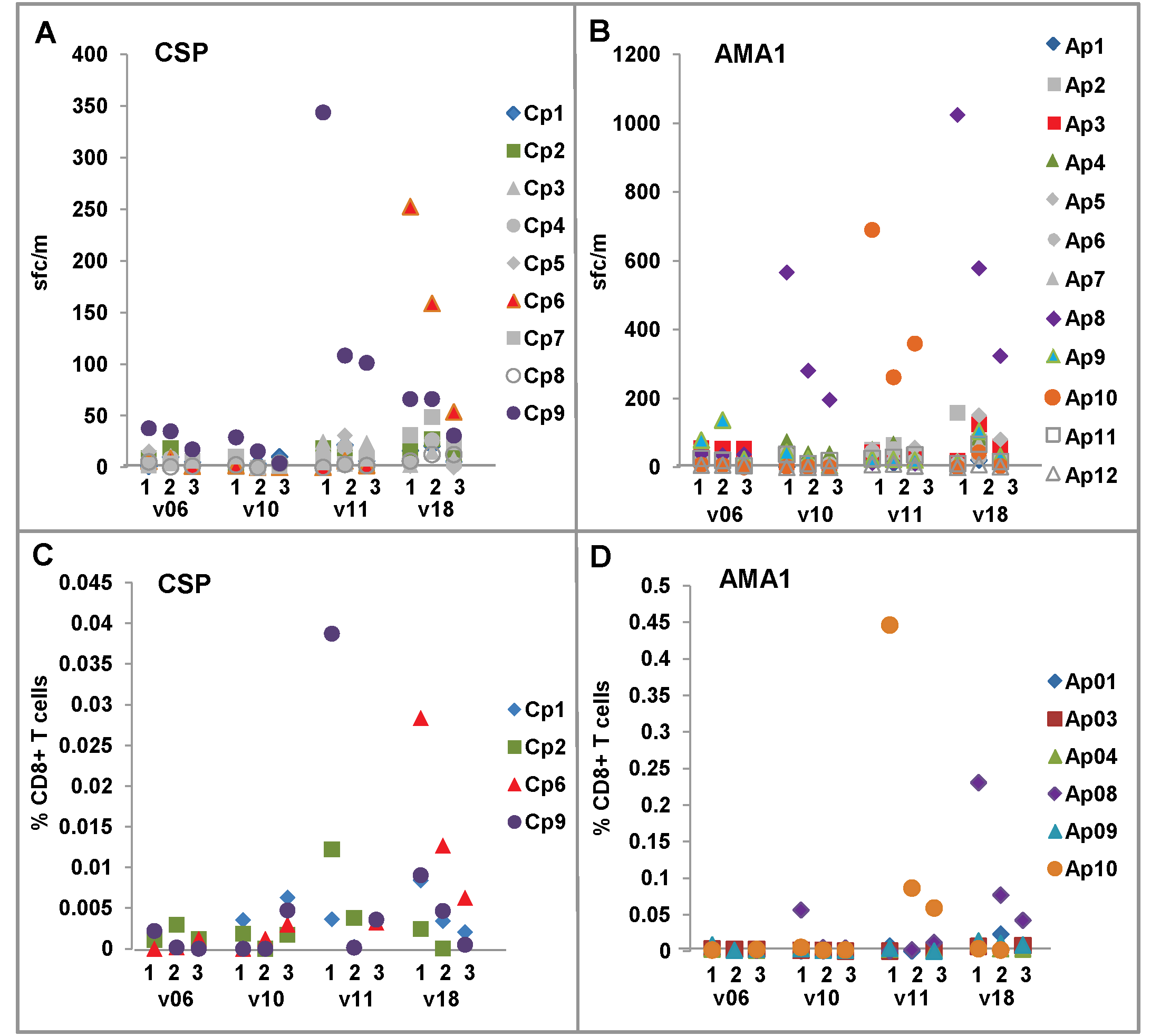

Supplement: Figure S6 — DNA/Ad: Pre- and post-challenge T cell responses to CSP and AMA1 peptide pools. The T-cell activities of protected volunteers v06, v10, v11 and v18 to CSP and AMA1 peptide pools were measured 22/23 days after the Ad boost, 5/6 days before malaria challenge (1), 28 days (2) and 84 days (3) after malaria challenge. Panels A and B: ELISpot activities; Panels C and D: CD8+ T cell EM IFN-γ activities. With ELISpot and CD8+ T cell EM IFN-γ activities at 28 days after challenge of protected volunteer v11 fell with Cp9, v11 fell with Cp6, v10 and v18 fell with Ap8 and v11 fell with Ap10, and all activities generally declined further by 84 days after challenge. In contrast ELISpot activities of non-protected volunteers all rose at 28 days after challenge, and then declined by 84 days after challenge (not shown); CD8+ T cell EM IFN-γ activities remained negative after challenge. Therefore, after challenge ELISpot pool-specific activities and CD8+ T cell EM IFN-γ activities, although lower, maintained the same specificity as before challenge, with the exception of v11 CD8+ T cell EM IFN-γ activity with Cp9 that fell below the positive cut off. (TIFF) [file pone.0106241.s006.tiff]
