# Supplementary material for: Sterile Immunity to Malaria after DNA Prime/Adenovirus Boost Immunization Is Associated with Effector Memory CD8+T Cells Targeting AMA1 Class I Epitopes
Source: PLoS One. 2014 Sep 11;9(9):e106241. doi: 10.1371/journal.pone.0106241 (PMC4161338; doi:10.1371/journal.pone.0106241)
Supplement: Table S1 — Predicted class 1-restricted epitopes within CSP and AMA1 peptide pools predominantly recognized by protected and non-protected volunteers. (DOCX) [file pone.0106241.s007.docx]

**Table S1. Predicted class 1-restricted epitopes within CSP and AMA1 peptide pools predominantly recognized by protected and non-protected volunteers**

| **Pool** | **Vol.** | **Status** | **HLA** | **Epitope** | **IC_50_ nM** | **Restriction** | **Supertype** | **Peptide** |
| --- | --- | --- | --- | --- | --- | --- | --- | --- |
| **Cp6** | v18 | Protected | A*02:01/A*02:05  B*44:02/B*58:01 | IQNSLSTEW  YLNKIQNSL | 88  29 | B*58:01  A*02:01 | B58  A02 | C48  C47/48 |
| **Cp9** | V11 | Protected | A*11:01/A*68:01  B*50:01/B*55:01 | DYANDIEKK | 371 | A*68:01 | A03 | C58 |
|  | v12 | NP | A*01:01/A*02:01  B*08:01/B*44:02 | GLIMVLSFL | 40 | A*02:01 | A02 | C64/65 |
|  | v15 | NP | A*29:02/A*30:02  B*15:01/B*57:01 | KMEKCSSVF  LIMVLSFLF | 44  116 | B*15:01  B*15:01 | B62  B62 | C60/61  C64/65 |
| **Ap8** | V18 | Protected | A*02:01/A*02:05  B*44:02/B*58:01 | KSHGKGYNW  FLPTGAFKA  CLINNSSYI  SMIKSAFLPT | 21  33  116  205 | B*58:01  A*02:01  A*02:01  A*02:01 | B58  A02  A02  A02 | A97/98  A94  A104  A93 |
|  | V10 | Protected | A*01:01/A*26:01  B*44:02/B*57:01 | KSHGKGYNW | 43 | B*57:01 | B58 | A97/98 |
|  | v15 | NP | A*29:02/A*30:02  B*15:01/B*57:01 | KSHGKGYNW  HGKGYNWGNY  SAFLPTGAF | 43  74  141 | B*57:01  A*30:02  B*15:01 | B58  A01  B62 | A97/98  A99  A94/95 |
|  | V156^2^ | NP | A*03:01/A*29:02  B*15:03/B*58:02 | FKNKNASMI  SAFLPTGAFK  KSHGKGYNW  FKADRYKSH  HGKGYNWGNY | 15  31  26^1^  150  334 | B*15:03  A*03:01  B*58:02^1^  B*15:03  A*29:02 | B27  A03  B58  B27  A01/A24 | A92  A94/95  A97/98  A96/97  A98/99 |
|  | V194^2^ | Partially protected | A*32:01/A*68:01  B*14:01/B*58:01 | SAFLPTGAFK  GAFKADRYK  KSHGKGYNW | 8  242  21 | A*68:01  A*68:01  B*58:01 | A03  A03  B58 | A94/95  A96  A97/98 |
| Ap10 | v11 | Protected | A*11:01/A*68:01  B*50:01/B*55:01 | STCRFFVCK  EVVVKEEYK  EMVSNSTCR  VTSNNEVVVK  FVCKCVERR  FISDDKDSLK  ISDDKDSLK | 7  7  11  20  30  38  260 | A*11:01  A*68:01  A*68:01  A*11:01  A*68:01  A*68:01  A*11:01 | A03  A03  A03  A03  A03  A03  A03 | A125  A130  A123/124  A129  A126/127  A120  A121 |
|  | v03 | NP | A*02:01/A*02:01  B*14:02/B*18:01 | NEVVVKEEY  KIIAPRIFI | 12  443 | B*18:01  A*02:01 | B44  A02 | A130  A118/119 |

Class 1-restricted 8-10mer epitopes with CSP and AMA1 peptide pools, predominantly recognized by protected and non-protected volunteers who had positive activities to each peptide pool (Table 2), were predicted using NetMHC. The binding affinities (IC_50_ 500 nM) are shown as nanomolars (nM). The HLA-restrictions are assigned to HLA supertypes (reference 41). Location of predicted epitopes is shown as the 15mer peptide contained within that peptide pool. Non-protected v156 from the AdCA trial is included as T cell activities were not consistent with protection. NP=Not Protected. ^1^Prediction is from SYFPEITHI (reference 40). ^2^Volunteers are from the AdCA trial.
